# Design and performance assessment of a pelleting machine for sustainable biomass pellet fuel production from plant residues

**DOI:** 10.1038/s41598-025-93058-6

**Published:** 2025-04-15

**Authors:** Mohamed M. Ibrahim, Samy M. Younis, Abd El-Aal Z. Taieb, Badr Azzam, Mohamed Ghonimy

**Affiliations:** 1 Department of Agricultural Engineering, Faculty of Agriculture, Cairo University, Giza, Egypt; 2 Department of Mechanical Engineering, College of Engineering, Taibah University, Al Madinah Al Munawwarah, Saudi Arabia; 3 Department of Agricultural and Biosystems Engineering, College of Agriculture and Food, Qassim University, P.O. Box 6622, 51452 Buraydah, Saudi Arabia

**Keywords:** Cotton stalks, Energy, Finite Element Analysis (*FEA*), Pellet fuels, Pelleting machine, Plant residues, Biofuels, Environmental sciences, Energy science and technology, Engineering

## Abstract

This study designs and develops an energy-efficient pelleting machine for crop residues, integrating the Finite Element Method (*FEM*) and experimental evaluations. Key performance parameters including machine productivity (*M*_*p*_), pellet length (*Pl*), particle density (*ρ*_*p*_), bulk density (*ρ*_*b*_), hardness resistance (*Hr*), shear stress (*τ*), and pellet durability (*Dp*) were analyzed under varying conditions of moisture content (*MC*), molasses content (*MLC*), particle size (*PS*), and main shaft rotating speed (*RS*). Results showed that increasing *MC*, *MLC*, *PS*, and *RS* significantly improved pellet quality and efficiency. Peak *Mp* (99.4 kg h^−1^) was achieved at 20% *MC*, 0.7 mm *PS*, and 100 rpm, while 15% *MLC* yielded the highest *ρ*_*p*_ (1147 kg m^−3^), *ρ*_*b*_ (610 kg m^−3^), *Hr* (447 N), and *τ* (3.6 MPa). Pellet durability reached 94%, highlighting the molasses’ superior binding properties. Heatmap analysis confirmed strong correlations between *MLC* and critical pellet properties. The energy-efficient process consumed 128.45 kW h ton^−1^, only 2.8% of the energy potential of cotton stalks, ensuring sustainability. This study introduces a novel approach to enhancing pellet quality while minimizing synthetic additives, demonstrating advancements in process efficiency. Future research should investigate advanced binder formulations, process automation, and the long-term stability of biomass pellets under various storage conditions.

**Supplementary Information:**

The online version contains supplementary material available at 10.1038/s41598-025-93058-6.

## Introduction

The global demand for energy is increasing due to technological advancements, population growth, and evolving lifestyles, exerting pressure on conventional energy resources^1^. Although renewable energy technologies are advancing, achieving full economic feasibility as a substitute for traditional energy remains challenging. This situation underscores the need for integrated energy policies prioritizing sustainable resource management alongside socioeconomic development^2^.

Agricultural residues such as cotton stalks, rice straw, and corn stalks offer a promising alternative for renewable energy production because their energy content is comparable to that of coal^3^. However, their low density and high moisture content present challenges for handling, storage, and transportation. Converting these residues into compact forms such as briquettes and pellets effectively mitigates these issues. This study focuses on cotton stalks as the primary biomass feedstock due to their abundance, renewable nature, and underutilization in many agricultural regions. Their utilization in pellet production not only promotes sustainable biomass use but also supports waste valorization and a circular bioeconomy. Additionally, cotton stalks possess favorable physical and chemical properties, particularly adequate lignin content, which enhances pellet binding and durability, making them an efficient raw material for biofuel production.

Pelleting compresses loose organic materials into dense, uniform shapes, providing a renewable alternative to conventional fuels like firewood or coal especially in rural areas where local resources and simple engineering solutions can be employed^4^. Research shows that densification can boost material density significantly (from around 100 kg m^−3^ to over 1000 kg m^−3^), thereby enhancing fuel usability while requiring minimal energy relative to the biomass’s energy content^5,6^. Moreover, this process reduces transportation costs and improves storage efficiency^7^, while also lowering greenhouse gas emissions by lessening reliance on fossil fuels and curtailing the environmental impacts of traditional residue disposal methods such as open burning^8^.

Advances in pelleting technology such as the use of additives like molasses to improve binding have further enhanced the structural integrity and combustion characteristics of the briquettes^9^. Additionally, the design and development of efficient pelleting machines are crucial to fully leveraging the potential of agricultural residues. Modern engineering tools like the Finite Element Method (*FEM*) are used to optimize machine design for durability and efficiency, predicting stress distribution and material behavior to identify potential weaknesses before manufacturing^10^. Performance evaluations under various conditions (including moisture content, particle size, and machine speed) indicate that productivity can range from 50 to 100 kg h^−1^^11^.

Despite significant advancements in biomass pelleting technologies, a notable research gap remains in integrating advanced numerical simulation techniques such as the Finite Element Method (*FEM*) with practical machine design to effectively address the unique challenges posed by crop residues. Addressing this gap is crucial to developing efficient and sustainable pelleting machines that can effectively overcome the inherent challenges of crop residue processing. Thus, the present work aims to design and develop a machine capable of effectively pelleting crop residues. The study employs the Finite Element Method as a core tool in the design process and evaluates the machine’s performance to ensure it meets the desired compaction outcomes.

## Materials and methods

The experiment was conducted at the Faculty of Agriculture, Cairo University, Egypt to design and evaluate the performance of a pelleting machine intended for the sustainable production of biomass pellet fuel using plant residues.

### Cotton stalks preparation

Cotton stalks (“*Gossypium hirsitum L.*”, 145 cm average length, 8–16 mm bottom diameter, 4–8 mm top diameter) were obtained from the farm of Faculty of Agriculture, Cairo University, Giza, Egypt and characterized (Supplementary material, Table S1). Moisture content (7% d.b) was determined using the ASAE S358.2 standard, oven-drying 25 g samples at 105 °C for 24 h, with rapid checks via a moisture meter. Stalks were chopped using a Vermeer machine with a flywheel drum cutter, tested for size, and ground with a Wiley Cutting Mill (990 rpm, 8 hammers). Ground material was sieved (2, 1.4, 0.7 mm openings) per ANSI/ASAE S319.3 standards. This process ensured uniform material for compaction and further experiments. Additional experimental details can be found in the supplementary material (S.1.2, S.1.3 and S.1.4).

### Design of the pelleting machine

**Fig. 1:**
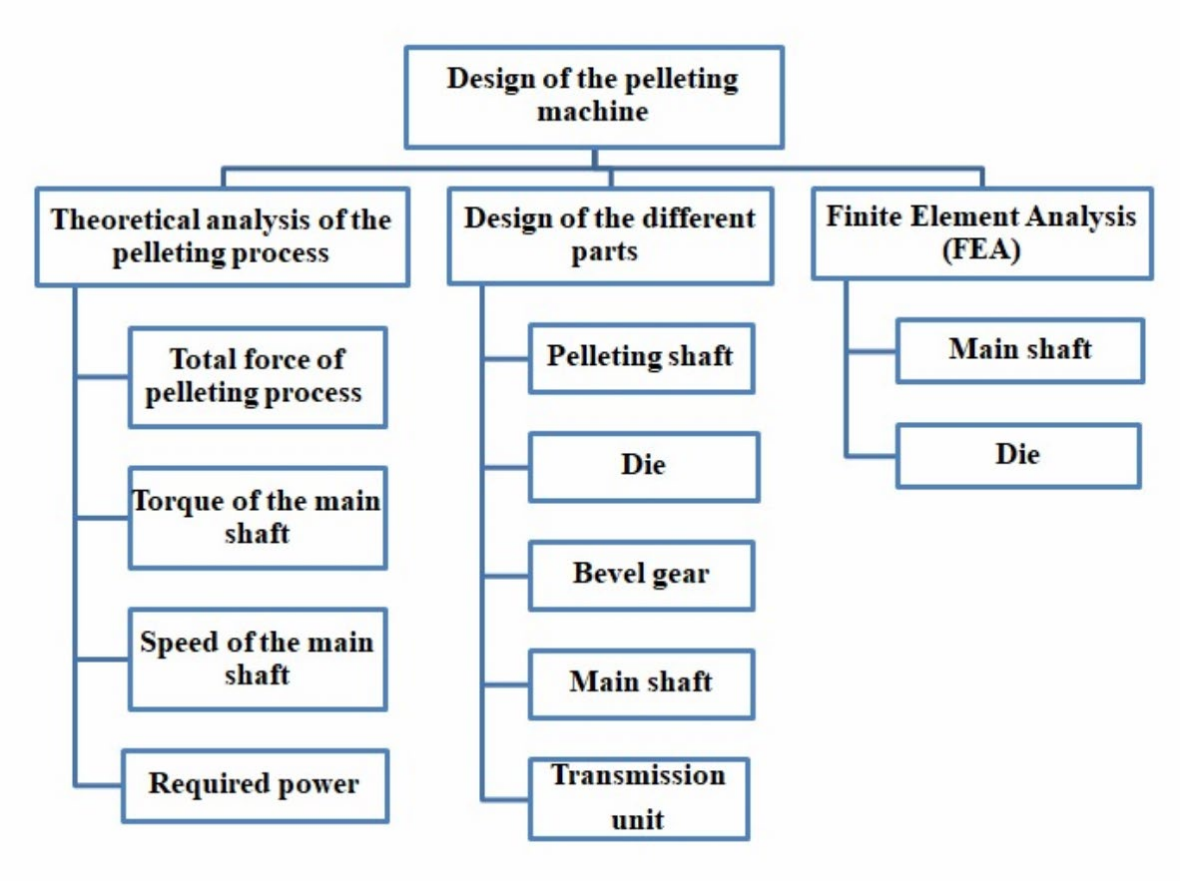
The design procedures of the pelleting machine.

Figure 1 illustrates the structured design process of the pelleting machine, divided into Theoretical Analysis, Component Design, and Finite Element Analysis (*FEA*). Theoretical analysis optimizes efficiency by evaluating force, torque, speed, and power. Component design focuses on key parts like the pelleting shaft, die, bevel gear, main shaft, and transmission unit. *FEA* assesses the strength and durability of the main shaft and die under operational stresses. This integrated approach ensures improved performance, durability, and efficiency. A pellet machine was designed to address the identified problem, featuring a fixed-die system with components including a hopper, twin forming roller, fixed die, cutting knife, transmission unit, electric motor, and frame. Biomass dust is fed through the hopper multiple times, where rotating rollers compress and shape it against the fixed dies. The centrifugal roller motion generates frictional heat at the dies, eliminating the need for additional heating, while softening and compacting the material to produce solid pellets. This design ensures effective pressure application to push the material through the die holes efficiently. The full dataset is available in the “Supplementary Material”.

#### Theoretical analysis of the pelleting process

**Fig. 2:**
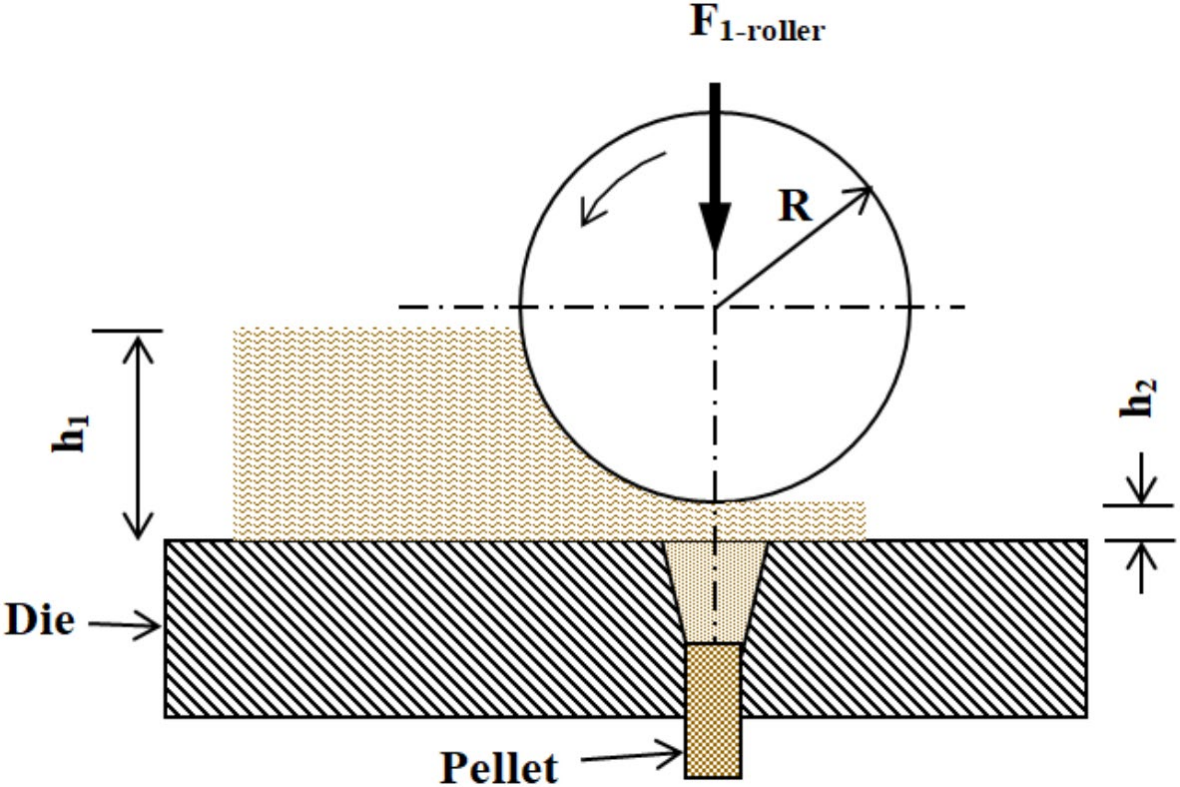
Diagramtic sketch of the pelleting process.

The pelleting process (Fig. 2) involves compressing raw material using a rotating roller (*R*), which applies a downward force (*F*_1_-*roller*) onto the material layer (*h*_1_) over the stationary die. As the roller rotates, it compacts the material into the die holes under high pressure, forming cylindrical pellets of length *h*_2_. This enhances particle adhesion, improving pellet durability and quality. The process was simulated in two stages: rolling and extrusion, with calculations performed to determine the required power. A single roller was considered for analysis, ensuring efficient high-density pellet production with consistent mechanical properties.

##### Coefficient of friction between the ground material and roller

For achieving a complete rolling process of the ground material, the following relation must be satisfied, Semenov et al.^12^.

1 $$\mu \ge \frac{{\sqrt {R \Delta h} }}{{R - \frac{\Delta h}{2}}}$$

where *µ* is the friction coefficient between the ground material and roller or between the ground material and die. It ranges between 0.68 and 0.86; $\Delta h = h_{1} - h_{2}$; *h*_1_ is the height of the ground material in front of the roller, mm; *h*_2_ is the height of the ground material after passing under the roller, mm; and *R* is roller radius, mm. In Eq. (1), when $\mu$ equals 0.68 and 0.86 the $\Delta$*h*_*min*_ equals 18 and 26.26 mm, respectively.

##### Rolling force (***F***_***roll***_)

The rolling force of the ground material can be determined from the following equation, Semenov et al.^12^.

2 $$F_{roll} = 1.38\sigma_{{f_{m} }} w\sqrt {R\Delta h}$$

where *w* is the roller width, 60 mm; $\sigma_{{f_{m} }}$ is the mean flow stress of the ground material = $\frac{{\sigma_{{f_{1} }} + \sigma_{{f_{2} }} }}{2}$; $\sigma_{{f_{1} }}$ is the flow stress before rolling (in front of roller), it can be neglected because the ground material is loose; $\sigma_{{f_{2} }}$ is the flow stress after rolling (after the roller). $\sigma_{{f_{2} }}$ = $\frac{F}{A}$ = 0.9947 N mm^−2^; and *A* is the area of the pellet that used in the test mm^2^. The calculation of the rolling force (***F***_***roll***_) yields a rolling force of value 1575 N.

##### Extrusion force (***F***_***ext***_)

**Fig. 3:**
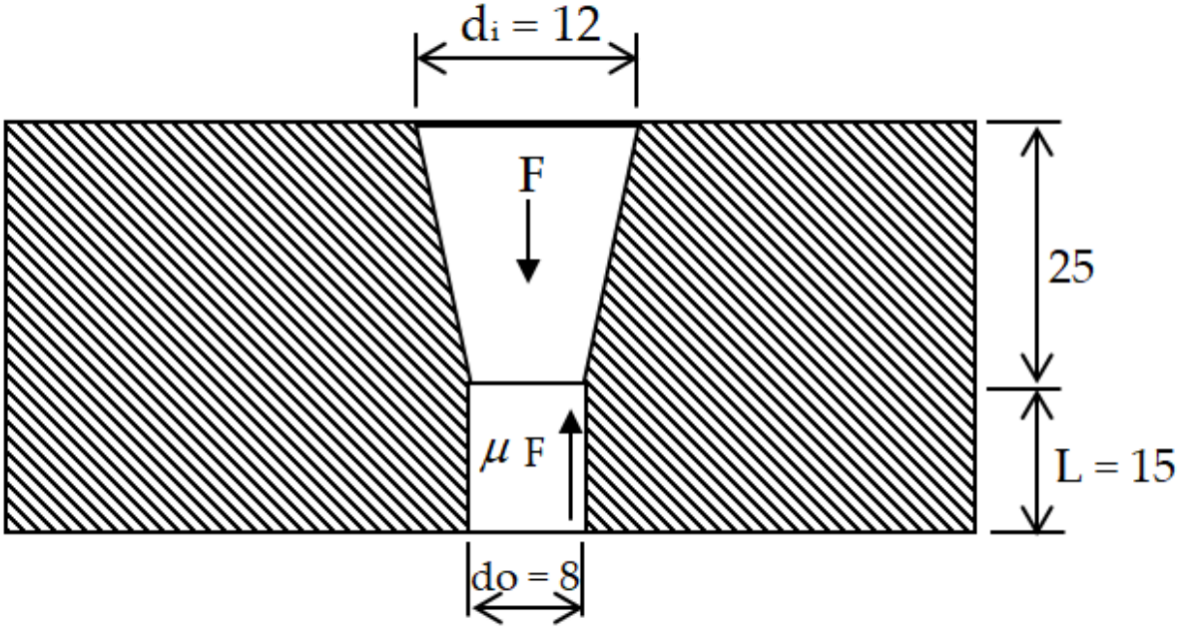
Extrusion Force of the pelleting process.

Extrusion force (*F*_*ext*_) of ground material into the orifice can be computed from the following relation as shown in Fig. 3, Semenov et al.^12^.

3 $$F_{ext} = P_{ext} Ai$$

where *Ai* is inlet area of orifice before extrusion, mm^2^; *Ao* is outlet area of orifice after extrusion, mm^2^ *P*_*ext*_ is the extrusion pressure $= \sigma_{f2} \ln O_{ar}$, Pa; $\sigma_{y}$ is the yield strength of the extruded agricultural ground material, $\sigma_{{f_{2} }}$ = 0.9947 N mm^−2^; and *O*_*ar*_ is orifice area ratio $= \frac{{A_{i} }}{{A_{o} }} = \frac{{d_{i}^{2} }}{{d_{o}^{2} }} = 2.25$. Thus, the extrusion pressure can be calculated and then the extrusion force can be determined as follows: *P* = 0.8066 N mm^−2^, *F*_*ext*_ = *P*_*ext*_ *Ai* = 0.3184 N mm^−2^ and *F*_*ext*_ = 91.18 N.

##### Friction force (***F***_***die***_)

Another force that found in this process is force die friction that arisen when pellet moves through the hole of the die (the straight section of hole), this force die (*F*_*Die*_) can be calculated from the following equation:

4 $$\it \it \it \it \it \it F_{{{\text{Die}}}} =\upmu \;{\text{P}}\;{\text{A}}_{{\text{s}}} =\upmu \;{\text{P}}\;\uppi \;{\text{d}}_{o} \;L_{h}$$

where *F*_*die*_ is friction force due to the moving the pellet through hole, N; $\mu$ is the coefficient of friction between the material and the surface, 0.8; *P*_*ext*_ is the applied pressure, *P*_*ext*_ = 0.3184N mm^−2^; *A*_*S*_ is the orifice surface area = $\pi d_{o} L_{h}$; *d*_*o*_ is the diameter of the hole, 8 mm; and *L*_*h*_ is length of the straight section of the hole, 15 mm. The force resists the die friction can be calculated to be 96 N. From the previous, it can be concluded as follows: *F*_*roll*_ = 1302.62 N, *F*_*ext*_ = 91.18 N and *F*_*die*_ = 96 N.

##### Total force of one roller for pelleting process (*F*_1*-roller*_)

The total force of pelleting process is the sum of all forces generated: rolling, extrusion and friction forces of the die^13^. It can be represented by Eq. (5).

5 $$\it \it \it \it {\text{F}}_{{1{\text{ - roller}}}} = F_{roll} + {\text{N}}\left( {F_{ext} + F_{die} } \right)$$

where *F*_1*-roll*_ is the total force of the pelleting process for one roller, N; and *N* is the number of orifices that roller covers them simultaneously, *N* = 4. Thus, the total force of pelleting process for one roller equals 2051.34 N.

#### Torque of the machine main shaft

**Fig. 4:**
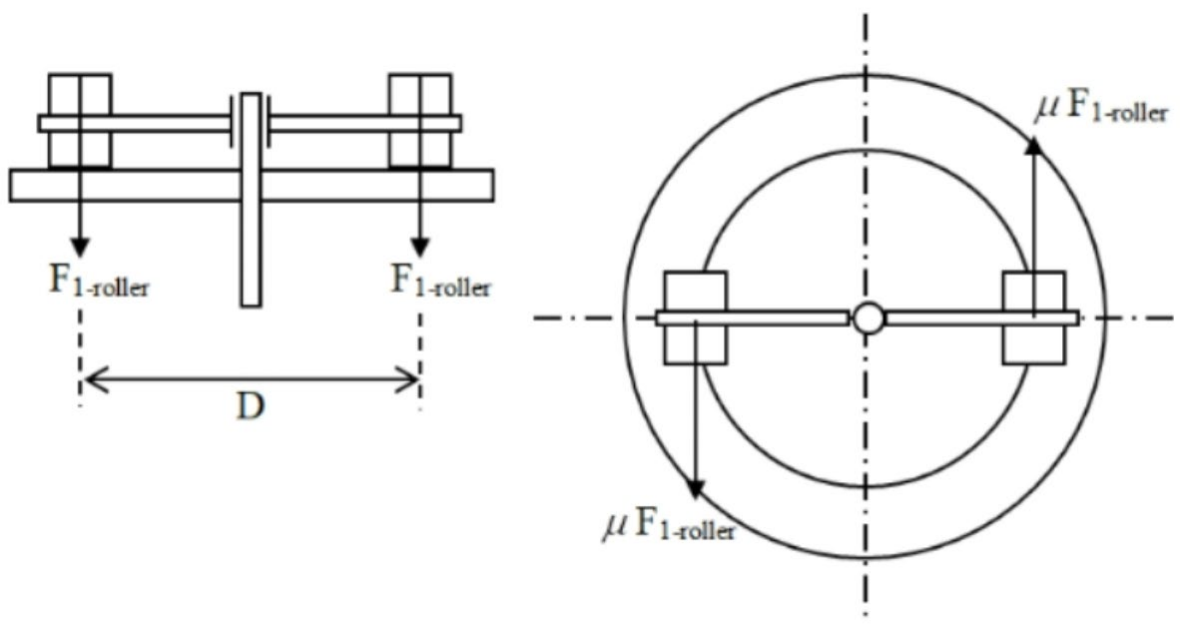
Die and rollers of the pelleting machine.

Torque (*T*) that applied on the main shaft to start the machine work was calculated as follows^14^, Fig. 4.

6 $$T = N_{roll} \times \mu_{roll} \times F_{1 - roll} \times \left( {D_{roller} /2} \right)$$

where *N*_*roll*_ is the number of the roller; $\mu_{roll}$ is coefficient of friction (rolling) between the ground material & die equals 0.6; *D*_*roller*_ is distance between rollers = 210 mm. The *T* for one roller equals 258.5 N m.

#### Speed of the main shaft of the machine

To get the right acting between the roller and the die the following relation should be achieved, Semenov et al.^12^.

7 $$\it \it \pi {\text{ D}}_{{{\text{roller}}}} \le { }P_{td} \left( {\text{Track of die}} \right)$$

where *Ptd* is the track of roller = $\frac{\pi }{2}D$_die_ (for two roller). The roller must at least revolve one revaluation in this track $\pi D_{roller} \le \frac{\pi }{2}D$_die_ or $D_{roller} \le \frac{1}{2}D$_die_. D_roller_ = 110 mm and *D*_*die*_ = 250 mm.

#### Required power (*RP*)

The required power can be calculated for pelleting process from Eq. (8).

8 $${\text{Power}} = T \times \omega$$

where *T* is the torque acting on the main shaft = 258.5 N m; $\omega$ is the angular velocity of the main shaft = $\frac{2\pi n}{{60}}$, rad s^−1^; and n is the rotational speed of the main shaft, rpm.

Recommended speeds of the main shaft can be found from the previous work in this field. The recommended peripheral speed of the roller is 2–3 m s^−1^^15^ which is equivalent to rotate speed of 150–230 rpm of the main shaft (2–3 m s^−1^ = $\pi$*D*_*die*_ *n*/60). So, the maximum speed equals 230 rpm, thus, $\omega =$ 24.07 rad s^−1^. Hence, the power required for the pelleting process will be 6.2 kW. Efficiency (*η*_*machine*_): 0.85, so the motor power = 7.5 kW.

### Design of the different parts of the pelleting machine

**Fig. 5:**
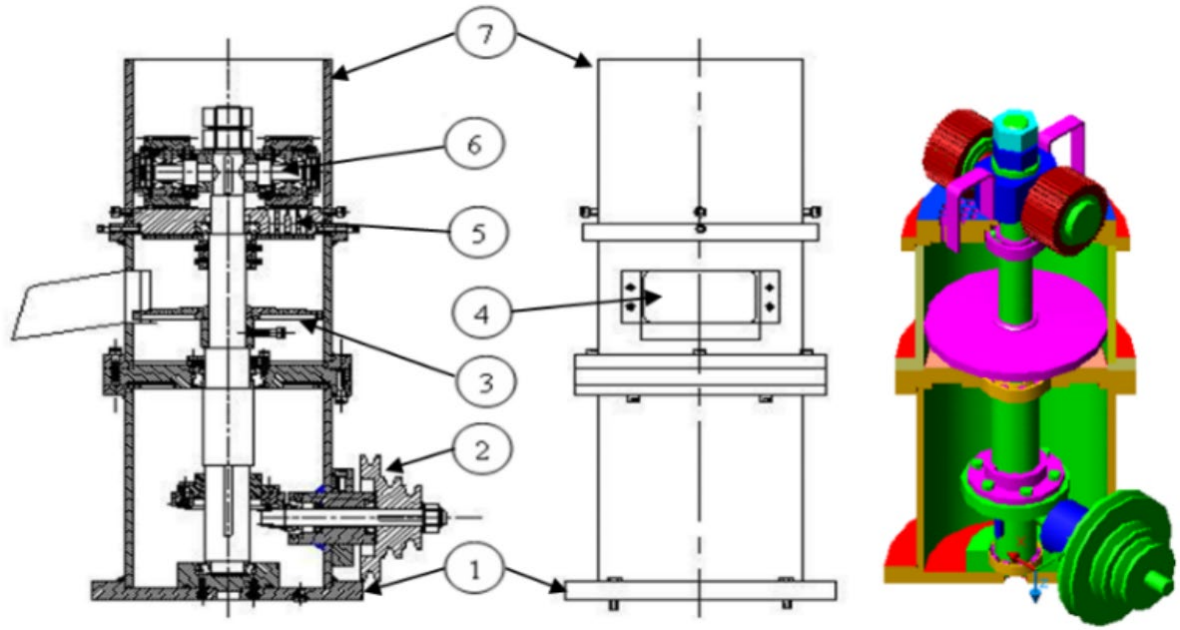
Schematic diagram of the pelleting machine: 1. Mounted base, 2. Transmission unit, 3. Collected tray, 4. Hopper, 5. Die, 6. Pelleted rollers, 7. Input orifice.

A sketch of the pelleting machine and its main components are presented in Fig. 5. The full dataset is available in the “Supplementary Material”.

#### Design of the pelleting shaft

The unit that carries the roller consists of two shafts and a head that relates to the main shaft. Each shaft is fitted to the head with a translation fit (Figure S8 in supplementary material). Therefore, the shaft was designed through the following procedures. Where maximum bending moment applying on shaft (Figure S9 in supplementary material).

9 $$M_{b} = F_{1 - roller} { } \cdot { }L_{s}$$

where *M*_*b*_ is the bending moment, N mm; and *L*_*s*_ is the distance from the roller to the head, mm. The ASME code equation for shafts subjected to torsion, bending, and axial loads by applying the maximum shear equation modified by introducing shock, fatigue and column factors (Equation S10, supplement material). According to the design calculations^16^, the shaft diameter is = 29.7 mm. The pelleting shaft must be equal or more than 30 mm for design safety.

#### Design of the die

**Fig. 6:**
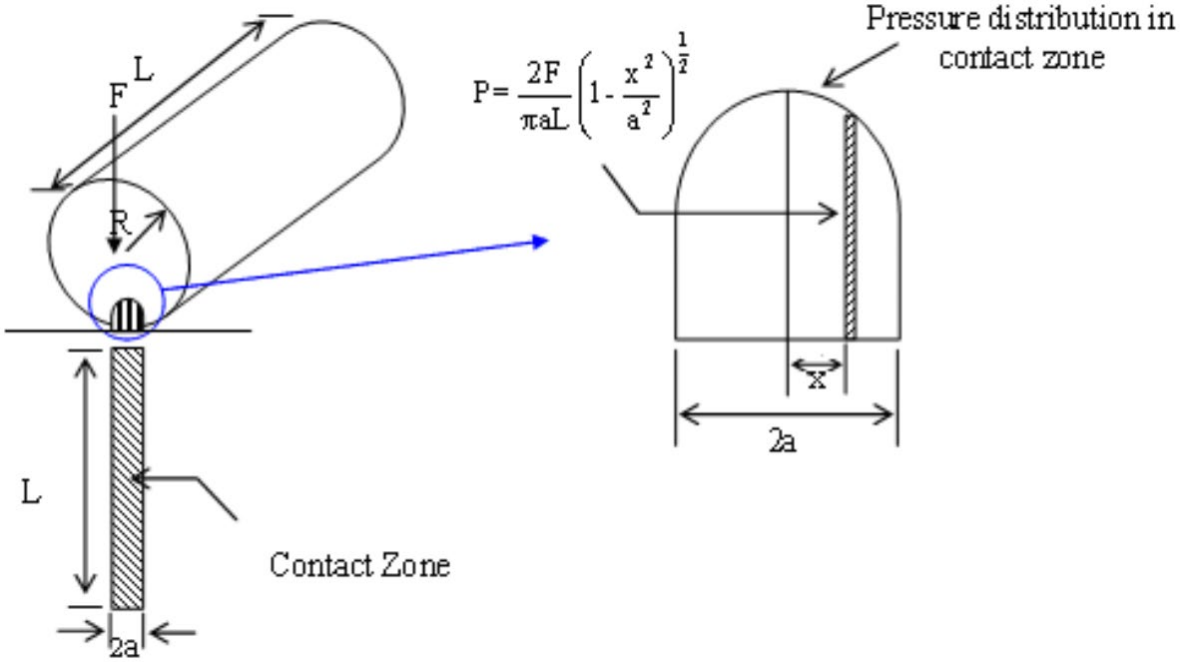
The contact stress of roller on the die plat (Hertzain stress).

The design of the die presented significant challenges due to the need to consider the geometry, contact, and friction between the fibrous biological material and the die steel, compounded by the complex mechanical behavior of the material being processed. A possible approach involves manufacturing various dies with differing tapered angles, lengths, and surface roughness. The roll press interaction with the die is analyzed using the Hertzian equation, which models the contact stress of a cylinder on a plane, as shown in Fig. 6.

The local pressure (*P*) in the contact area under an applied load (*F*) is defined as^17^.

10 $$\it {\text{P}} = \frac{{2{\text{F}}}}{{\uppi {\text{aL}}}}\left( {1 - \frac{{x^{2} }}{{a^{2} }}} \right)^{\frac{1}{2}} ,\quad {\text{a}} = \left[ {\frac{{4{\text{FR}}}}{{\uppi {\text{L}}}}\left( {\frac{{1 -\upgamma _{1}^{2} }}{{E_{1} }} + \frac{{1 -\upgamma _{2}^{2} }}{{E_{2} }}} \right)} \right]^{\frac{1}{2}}$$

where *a* represents half the contact width, mm*;* *L* is the roller length, mm; and *x* denotes the general position, *γ*_1_ is the Poisson’s ratio of the roll (cylinder), *γ*_2_ Poisson’s ratio of the plane, *E*_1_ is the Young’s modulus of the cylinder, *E*_2_ is the Young’s modulus of the plane.

The maximum contact stress (*P*_*max*_) between the roller and the die is calculated using Eq. (11)^17^ with parameters including the cylinder radius *R*, Poisson’s ratios ($\gamma_{1}$, $\gamma_{2}$), and Young’s moduli (*E*_1_, *E*_2_), where γ_2_ = 0.3 and *E*_2_ = 200 × 10^9^ N m^−2^. The maximum contact stress is determined to be *P*_*max*_ = 145.18 MPa.

11 $$\it P_{\max } = \frac{{2{\text{F}}}}{{\uppi {\text{aL}}}}$$

The annular distribution of the holes on the die as shown in the Figure S11 is 43, 37, 31, and 25 holes from the outer to inner diameter, respectively. The outer diameter of die and its thickness are 274 mm, 40 mm, respectively. The ratio between the opening area and the all area of the die is 0.367.

#### Design of the bevel gear

The bevel gear, specifically a hypoid bevel gear, is utilized to transfer power from the transmission unit to the main shaft. The kinematics of the gear involves an inverse relationship between the rotational speeds of the pinion (*n*_*p*_) and gear (*n*_*g*_) and the ratio of their diameters and number of teeth, expressed as^18^:

12 $$\frac{{n_{P} }}{{n_{g} }} = \frac{{N_{g} }}{{N_{P} }} = \frac{{D_{g} }}{{D_{P} }}$$

where *n*_*P*_, *n*_*g*_ are rotational speed of pinion and gear, respectively, *N*_*P*_, *N*_*g*_ are Number of teeth of pinion and gear, respectively, *D*_*P*_, *D*_*g*_ are pitch cycle diameter of pinion and gear, respectively.

The force analysis of the gear and pinion reveals that the total force (*W*) on the gear teeth can be resolved into tangential (*W*_*t*_), radial (*W*_*r*_), and axial (*W*_*a*_) components, For further analysis, refer to supplementary Figure S12 & Equation S14 (^19^, calculated as *W*_*t*_ = 2974.1 N, *W*_*r*_ = 1071.1 N, and *W*_*a*_ = 156.6 N.

#### Design of the machine main shaft

**Fig. 7:**
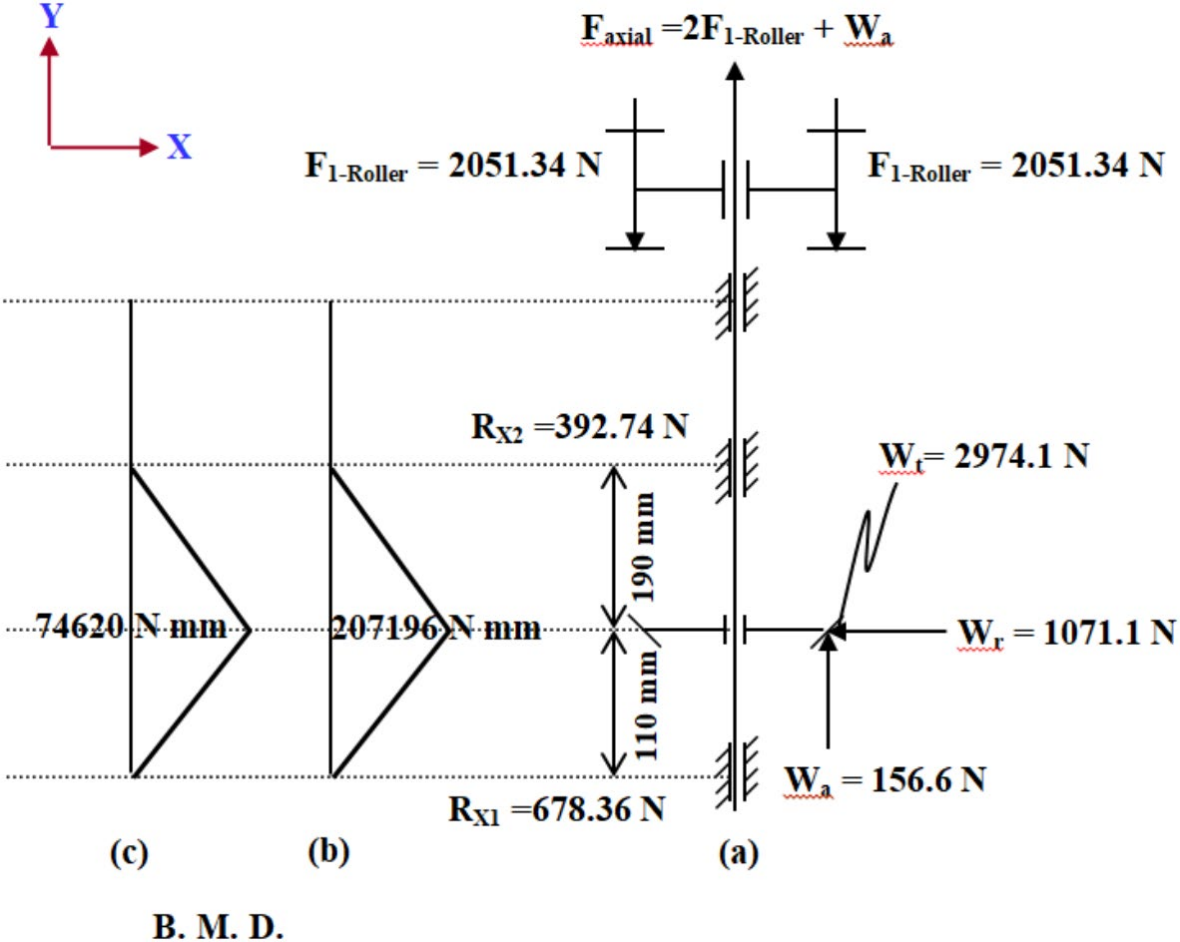
Loads acting on the main shaft and bending moments applied on it. (**a**) Shaft forces, (**b**) Bending moment around Z-axis, (**c**) Bending moment around X-axis.

The diameter of the transmission shaft was calculated according to ASME equation^20^. By applying all information in ASME code equation (Equation S2, supplement material), it can get the diameter of the shaft. Due to the bending moment, axial load, and the torque acting on the shaft as shown in Fig. 7, its design must account for these forces, requiring a diameter of at least 62 mm.

#### Transmission unit

**Fig. 8:**
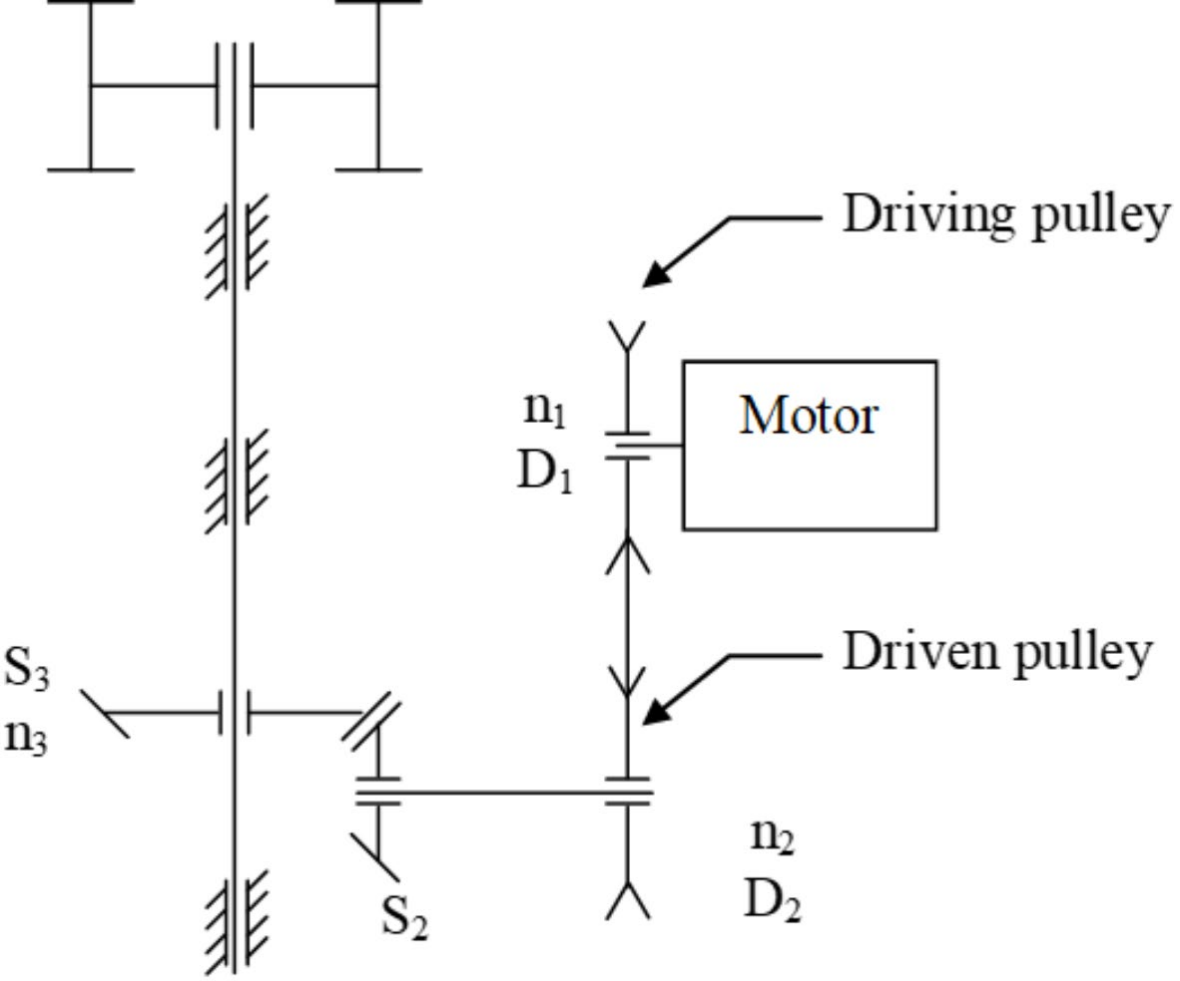
Power transmission system of the pelleting machine.

The power is transmitted from the electric motor to the machine via a belt-pulley system, as shown in Fig. 8.

The relationship between rotational speeds and pulley diameters is given by $\frac{{n_{1} }}{{n_{2} }} = \frac{{D_{2} }}{{D_{1} }}$ and $\frac{{n_{2} }}{{n_{3} }} = \frac{{s_{3} }}{{s_{2} }}$, where *n*_1_ is rotational speed of the electric motor = 1440 rpm, *n*_2_ is rotational speed of transmission shaft, n_3_ is rotational speed of the main shaft = (100, 80, 60) rpm, *D*_1_ is diameter of the motor pulley, *D*_2_ is diameter of the machine pulley, *S*_2_ is number of teeth in pinion, and *S*_3_ is Number of teeth in gear. The full dataset is available in the supplementary material (Section-S.2.3).

### Finite Element Analysis (*FEA*)

The Finite Element Analysis (*FEA*) technique, using COSMOS/M version 2.6, is a robust tool for predicting stresses and deformations in mechanical parts. Section S.3 (Supplementary material) illustrates the proposed model structure.

#### Numerical modeling of the main shaft

**Fig. 9:**
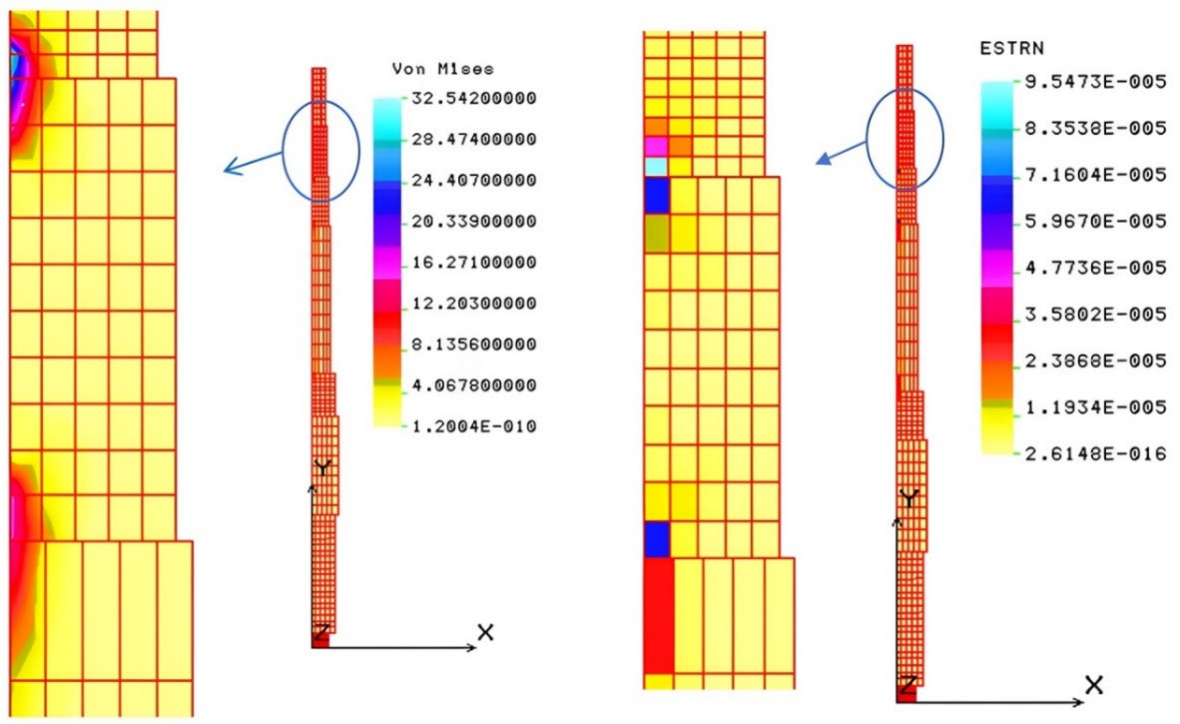
von-Mises stress (MPa) and Strain in the critical zone of the main shaft.

The main shaft of a pelleting machine was analyzed using Finite Element Analysis (*FEA*). The shaft receives power and transmits it to the rollers, with support from tapered roller bearings and a deep groove ball bearing. A PLANE 2D element with axisymmetry around the Y-axis was used, modeling the shaft in COSMOS/M as a half-section with 29 points, 40 curves, and 10 surfaces. Automated meshing created 500 elements and 660 nodes. Boundary conditions were set to restrict unnecessary degrees of freedom. Loading attributes included a tensile force from roller compaction and a torsional load applied. The analysis was conducted using a linear static approach to determine load distribution and stress behavior in the shaft. Additional information details are provided in the supplementary material (S.3.2). The von-Mises stress, resulting from tensile forces and torsional moments, ranges from 0.12 to 32.54 MPa, as shown in Fig. 9.

**Fig. 10:**
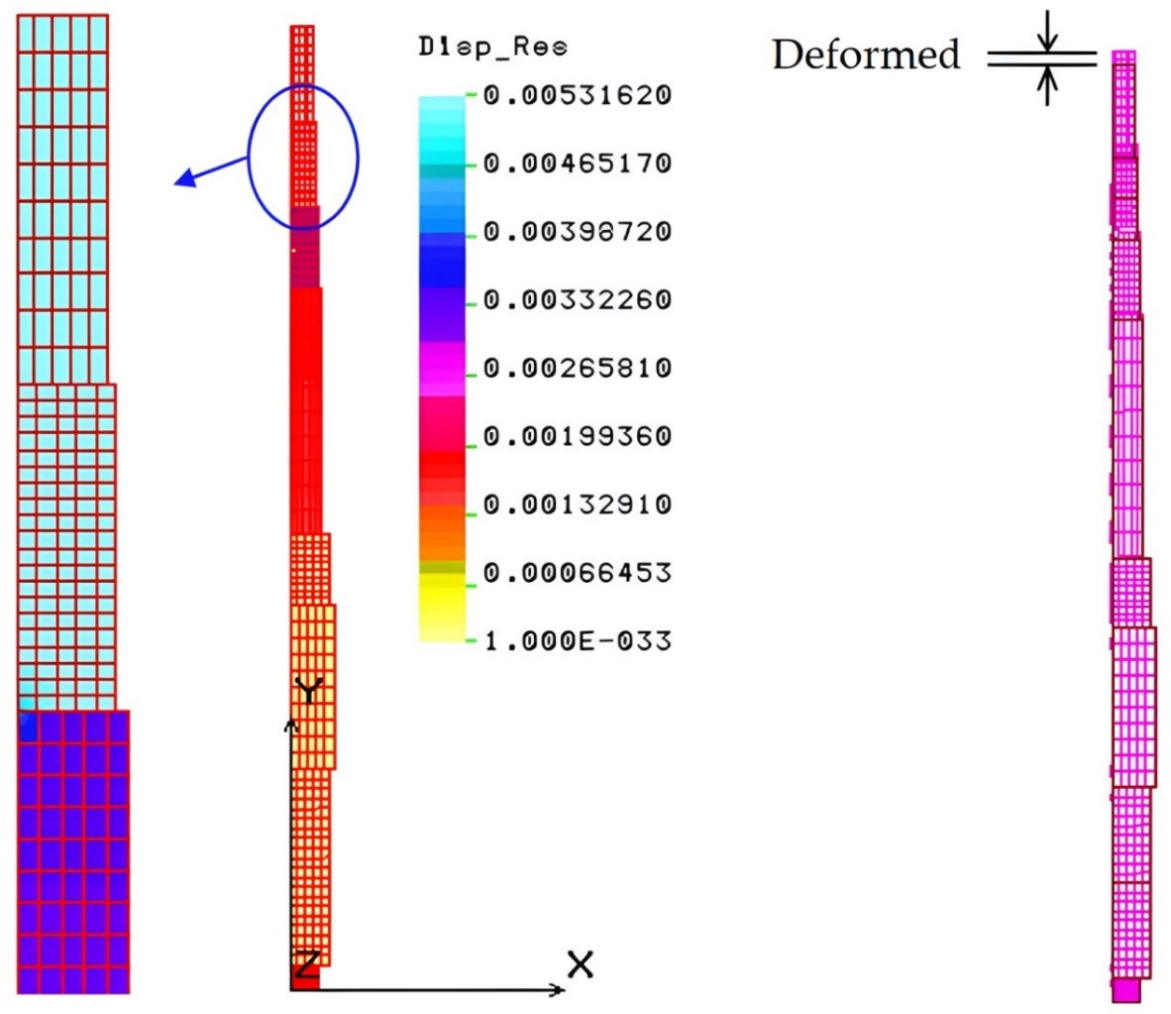
Displacement and deformation (mm) of the main shaft.

This stress is below the 40 MPa allowable limit, confirming the design safety of the main shaft. The maximum strain induced is 9.5 × 10^–5^, reflecting the shaft’s high rigidity, ensuring its toughness and stiffness. The maximum displacement is about 0.0053 mm, as shown in Fig. 10, further indicating the shaft’s rigidity.

#### Numerical analysis of the die

**Fig. 11:**
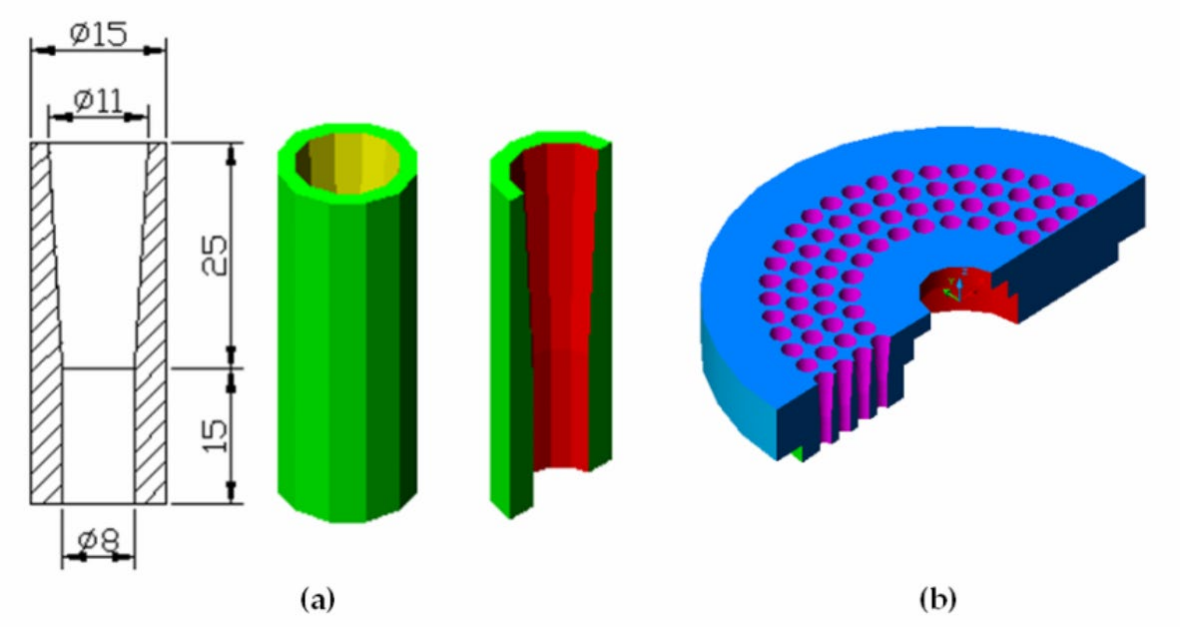
The one hole of the die (problem study). (**a**): The dimension of the problem study, (**b**): 3 D of the die.

The Finite Element Analysis (*FEA*) of the pelleting machine die is useful to verify theoretical design results and analyze stress and strain distributions. The die contains 136 extrusion holes, each with an 8 mm inside diameter, counterbored to 11 mm diameter and 25 mm depth. For simplicity, one extrusion hole is chosen for analysis. Each hole has an inside diameter (*ID*) of 8 mm, counterbored to 11 mm, and a height of 40 mm. The numerical analysis treats the chosen hole as a hollow cylinder with an inside diameter of 8 mm, counterbored to 11 mm and 25 mm deep, and an outside diameter of 15 mm, corresponding to the pitch diameter between holes, as shown in Fig. 11.

**Fig. 12:**
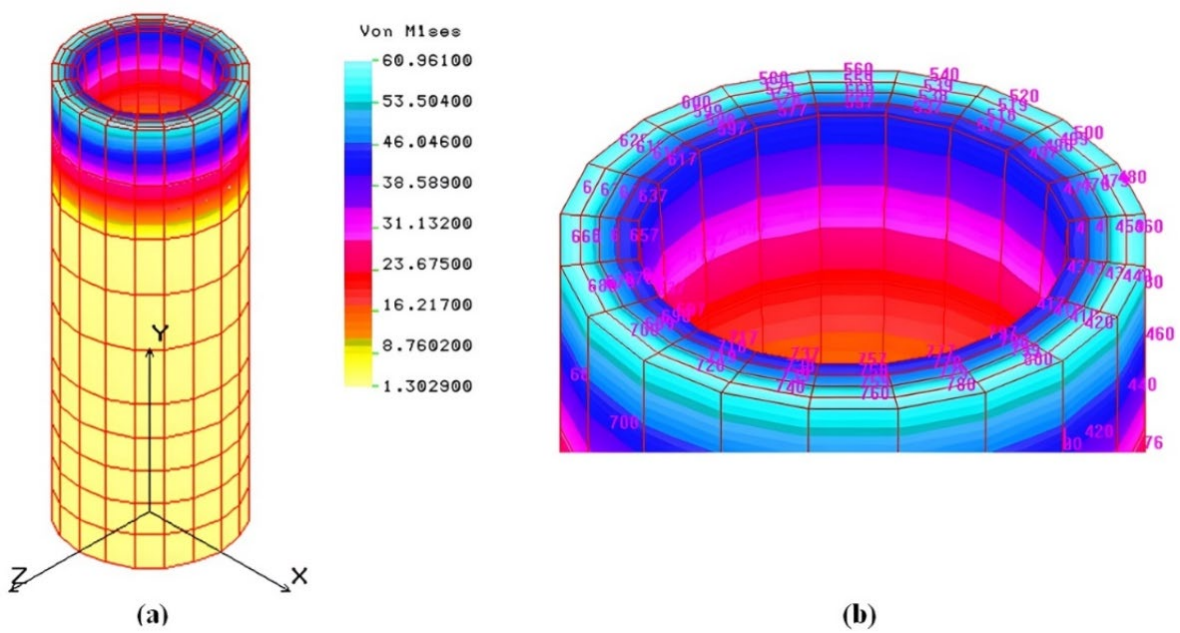
von-Mises stress (MPa) of one hole of the die: (**a**): 3 D; (**b**): Maximum stress part.

Finite Element Analysis (*FEA*) was applied to model the extrusion die hole using a SOLID element, an 8- to 20-node three-dimensional element for structural. The structural analysis considers three translational degrees of freedom per node, while the thermal module uses one degree of freedom per node for temperature representation. The die hole is modeled in COSMOS/M, represented in both 2D and 3D forms with 27 points, 52 curves, and 36 surfaces forming 8 volumes. Automatic meshing divides the model into 800 elements and 1410 nodes. Boundary conditions include displacement constraints are fully fixed with no translational or rotational degrees of freedom. Loading attributes involve applying pressure based on the Hertzian equation: 145.18 MPa on the hole’s top and additional pressure on internal surfaces. Additional information details are provided in the supplementary material (S.3.3). The finite element analysis reveals that the von-Mises stress varies between 1.3 MPa and 60.96 MPa due to the applied pressure from input to output power. Figure 12 shows the stress distribution along one hole of the die, with the maximum stress occurring around the outside of the hole but not exceeding 60.96 MPa, indicating the design’s safety, as the ultimate strength after hardening and tempering is 120 MPa.

**Fig. 13:**
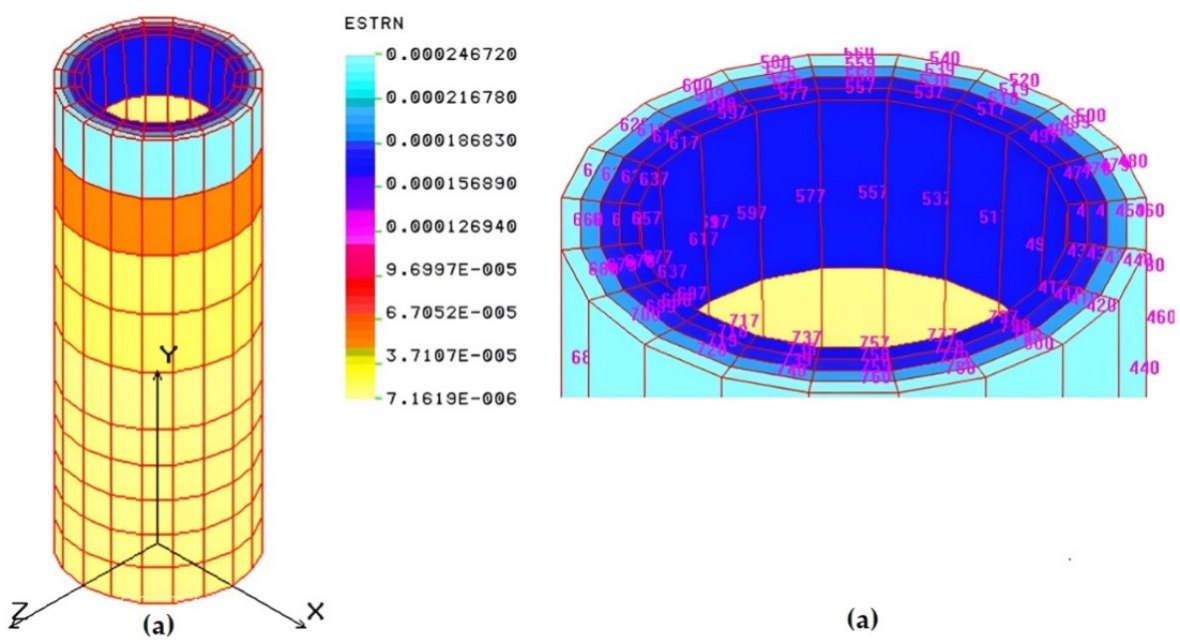
Strain distribution of one hole of the die: (**a**): 3 D; (**b**): Maximum strain part.

The maximum strain induced in the hole is 2.4 × 10^−4^, as shown in Fig. 13, reflecting the die’s high rigidity.

**Fig. 14:**
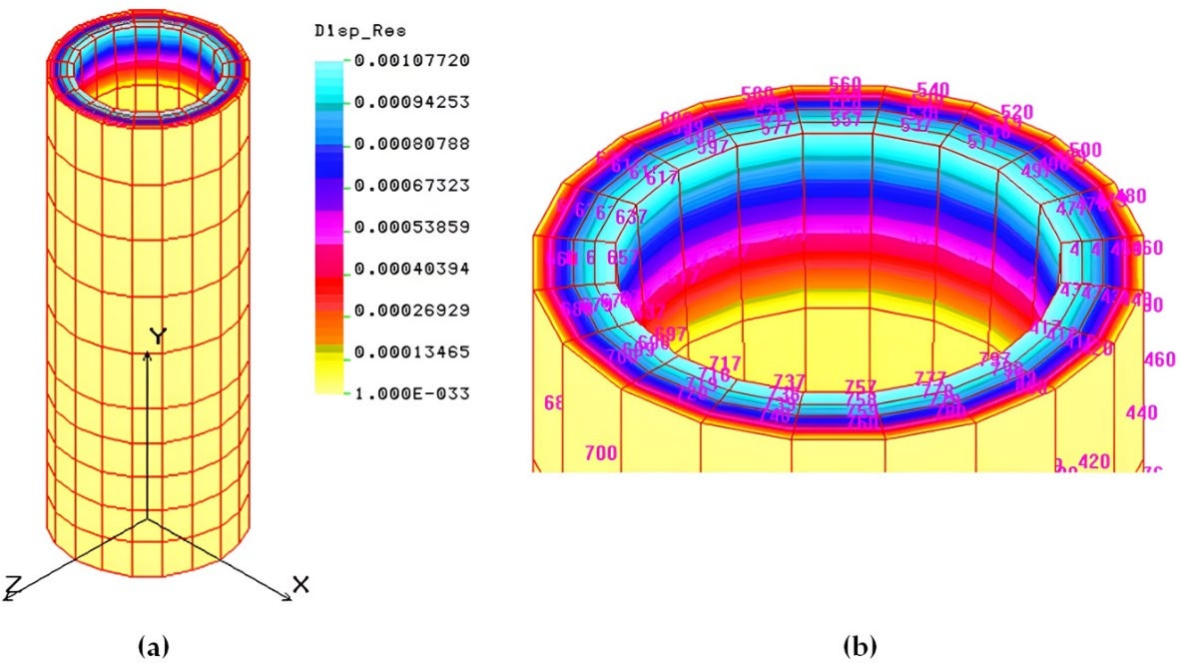
Displacement distribution (mm) of one hole of the die. (**a**): 3 D, (**c**): Maximum displacement par.

**Fig. 15:**
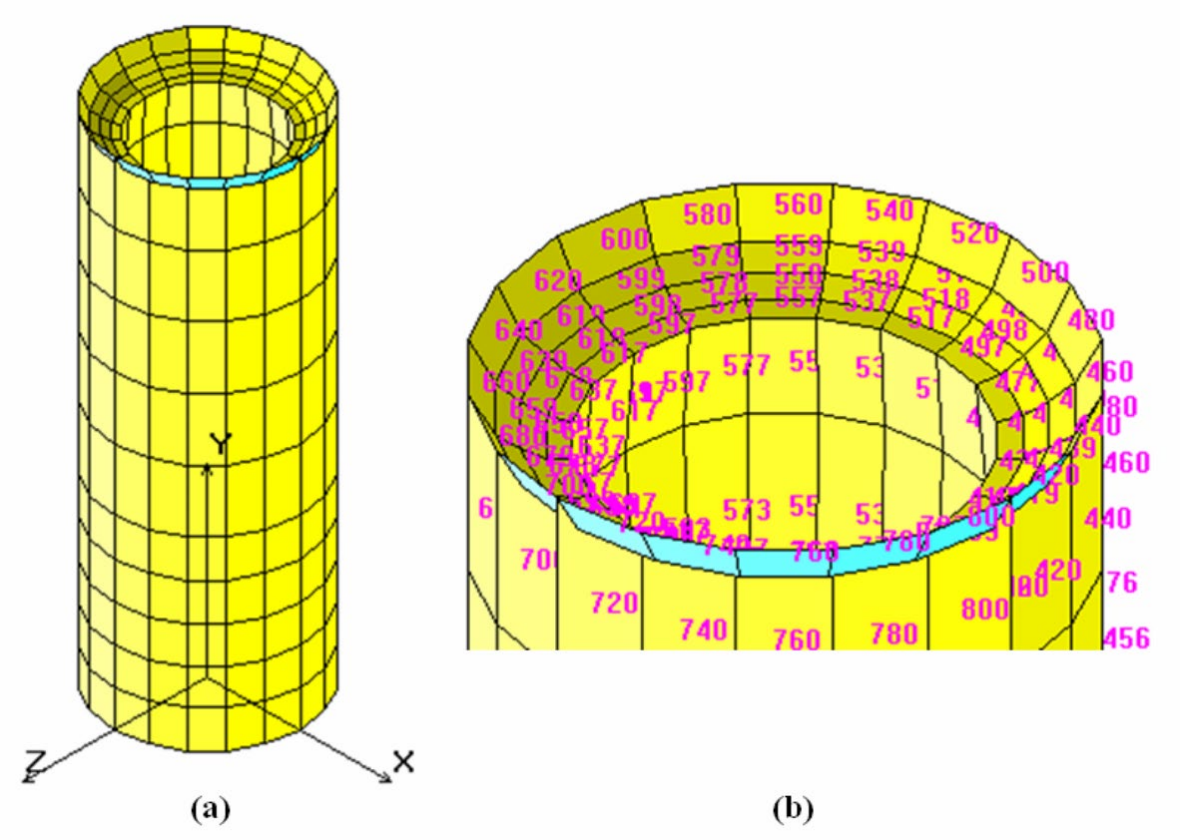
Deformed shape of one hole of the die.

The displacement of the hole due to the applied pressure is 0.001 mm, indicating low displacement and high rigidity, as shown in Figs. 14 and 15 displays the deformed shape of the hole.

### Assessment of machine performance

The ground cotton stalks were prepared and sprayed with water using a compression sprayer to adjust the *MC* by weight to 7, 15, and 20%. Alternatively, ground cotton stalks with a *MC* of 7% were mixed with molasses using a mixer to ensure proper blending, achieving a mixture with *MLC* of 5, 10, and 15%. Table 1 presents the experimental treatments for evaluating the pelleting machine. The selected values in Table 1 were chosen based on their influence on pellet quality and machine performance. The main shaft rotating speed (*RS*) was set at 60, 80, and 100 rpm to analyze its impact on material compression and pellet density, as higher speeds generally improve pellet compaction but may also increase energy consumption. The particle size (*PS*) varied at 0.7, 1.7, and 3.0 mm to evaluate how different material textures affect pellet durability and mechanical properties, with finer particles expected to enhance bonding and cohesion. The additive conditions included moisture content (*MC*) and molasses content (*MLC*) at different levels to assess their roles in improving pellet durability and hardness. Higher *MC* aids in binding but may reduce efficiency, while molasses enhance adhesion and increases pellet strength.

**Table 1:** Experimental treatment for the assessment.

Variables	Value
Main shaft rotating speed (*RS*), rpm	60, 80, 100
Particle size (*PS*), mm	0.7, 1.7, 3.0
Additive (*MC* or *MLC*)*, %	7 *MC*, 15 *MC*, 20 *MC,* 5 *MLC*,10 *MLC*,15 *MLC*

*MC, Moisture content; *MLC*, Molasses content.

#### Machine productivity (*Mp*)

The machine productivity was calculated using Eq. (13).

13 $$\it \it M_{p} = \frac{{{\text{Mw}}}}{t}$$

where *Mp* is the machine productivity, kg h^−1^; *Mw* is the pellet mass, kg; and *t* is the consumed time, h.

#### Pellet length (***P***_***l***_)

Average diameter of the pellet was 8 mm. The length of the pellet was measured using digital calliper. An important parameter in the context of pellet production is the length-to-diameter ratio, which is particularly relevant when using pneumatic feeding systems. This ratio is critical because even a single long pellet can block the transport pipe of such systems. According to Önorm^21^, the standards of the Austrian Pellets Association, this ratio should be less than 5 to ensure smooth and uninterrupted transport.

#### Pellet particle density (ρ*p*)

The *ρ*_*p*_ was determined using the direct measurement method in accordance with ASAE Standard S269.4^22^. It is noted that the measurements were conducted for 30 pellets, and the average value was calculated. The *ρ*_*p*_ was calculated from Eq. (14).

14 $$\rho_{p} = \frac{{P_{m} }}{{V_{p} }}$$

where $\rho_{p}$ is the particle density, kg m^−3^; *P*_*m*_ is the pellet particle mass, kg; *V*_*P*_ is the pellet particle volume of the = $\frac{\pi }{4}d^{2} L$, m^3^; *d* is the pellet diameter, m; and *L* is the pellet length, m.

#### Pellets bulk density (***ρ***_***b***_)

Bulk density was determined following ASAE Standard S269.4^22^ by filling a container (380 mm diameter, 495 mm high). Bulk density was calculated as the mass of the sample in the container divided by the container’s volume.

#### Pellet hardness resistance (*Hr*)

The pellet hardness resistance (*Hr*) was measured by loading them on the edge using flat plates. A digital force gauge (Universal testing machine, Instron—1000 N), according to Hassan et al.^23^ and Abdelhady et al.^24^, measured the force to rupture 50 pellets from each sample. Pellets were placed on a flat plate with the radial dimension aligned with the compressive force. A 50.8 mm diameter flat plate plunger was pressed onto the pellet, and the maximum force required to rupture the sample was determined according to Adapa et al.^25^ and McMullen et al.^26^.

#### Shear stress of pellet (τ)

**Fig. 16:**
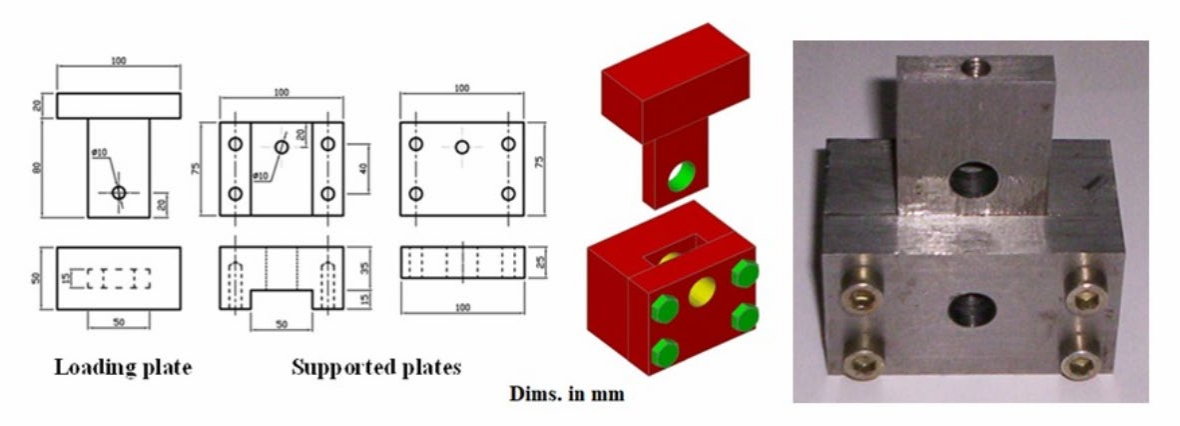
The double shear setup.

The shear stress of pellet (τ) was tested according to and ANSI/ASAE S459^27^. A shear box with a double shear setup (Fig. 16) was used, consisting of three hardened steel plates; two fixed and one sliding between them^28^.

The specimens were subjected to shear force, with the shear box connected to a steel rod linked to a digital force gauge (MARK-10, Model M3-10, USA) to measure the required shear force. The shear stress (*τ*, MPa) was calculated using the Eq. (15).

15 $$\tau = \frac{Fs}{{2A}}$$

where *F*_*s*_ is shearing force to cut the sample, N; *A* is sectional area of the pellet, mm^2^.

#### Pellet durability (*Dp*)

The mechanical durability of the pellets was assessed using a tester (MSD 71 4b, series No. 2016253214) in accordance with TS EN ISO 17831-1^29^. The resistance test device includes a 0.5 HP engine and a baffle (50 mm width, 230 mm length) positioned diagonally at the center of the cage. The mass of the pellet was calculated from Eq. (16).

16 $$\it {\text{Dp }}\left( \% \right) = \frac{{M_{A} }}{{M_{B} }} \times 100$$

where *M*_*A*_ is the mass of pellet after deformation, kg; and *M*_*B*_ is the mass of pellet before deformation, kg.

#### Gross calorific value (***GCV***) and pellets ash content 

Tests were conducted at the Biomass Labs, New and Renewable Energy Authority (*NREA*), Egypt. The calorific value of the pellets was measured using a bomb calorimeter (CAB003, Fisons Scientific Equipment, UK) per ASTM D2015-96, with combustion calibrated using benzoic acid tablets. Approximately 1 g of ground sample was ignited in oxygen, and the calorific value was expressed in kcal kg^−1^. Heating value was also measured using an IKA bomb calorimeter (C 240) following TS EN ISO 18125^30^. Pellets were ground to 1 mm, dried at 105 °C for 24 h, and analyzed based on temperature changes in the surrounding moisture.

Ash content was determined per ASTM D3174-97 by heating 1 g of ground sample (sieve #60) in a furnace, gradually raising the temperature to 700–750 °C, and maintaining it for two hours. The retained mass was expressed as percent ash content.

#### Energy consumption during pelleting process of cotton stalks

The mechanical energy required for chopping, grinding and pelleting processes was calculated based on the power rating of the machines and their operational productivity. Energy consumption (*CE*) was calculated from Eq. (17).

17 $$\it {\text{CE = }}\frac{{R_{p} }}{{M_{p} }}$$

where *CE* is the consumed energy, kW h ton^−1^; *Rp* is the required power, kW; and *Mp* is the machine productivity, kg h^−1^.

The labor energy was calculated using the assumption that a human can generate approximately 0.1 HP^31^. The labor energy is determined using Eq. (18).

18 $$\it {\text{EL}} = \frac{1000}{{1.34}} \times \frac{{L_{{p \times N_{L} }} }}{{M_{p} }}$$

where *EL* is labor energy, kW h ton^−1^; *N*_*L*_ is Number of labor; *M*_*p*_ is machine productivity, kg h^−1^; and *L*_*p*_ is the average power of the labor.

### Heat map analysis

A heat map was utilized to illustrate the strength and direction of correlations among variables such as moisture content, molasses content, particle size, shaft speed, and resulting pellet properties. This approach provides a visual summary of the data, allowing for a clearer interpretation of the relationships between key pelleting parameters. By presenting these correlations graphically, the heat map enhances the intuitive understanding of parameter interactions, highlighting significant trends that may not be immediately evident from numerical data alone.

## Results and discussion

The performance evaluation of the pelleting process involves analyzing key parameters that influence the quality and efficiency of pellet production. These parameters typically include pellet durability, density, energy consumption, and production rate. Understanding these factors is essential to optimize the process and enhance product quality.

### Machine productivity (***M***_***p***_)

**Fig. 17:**
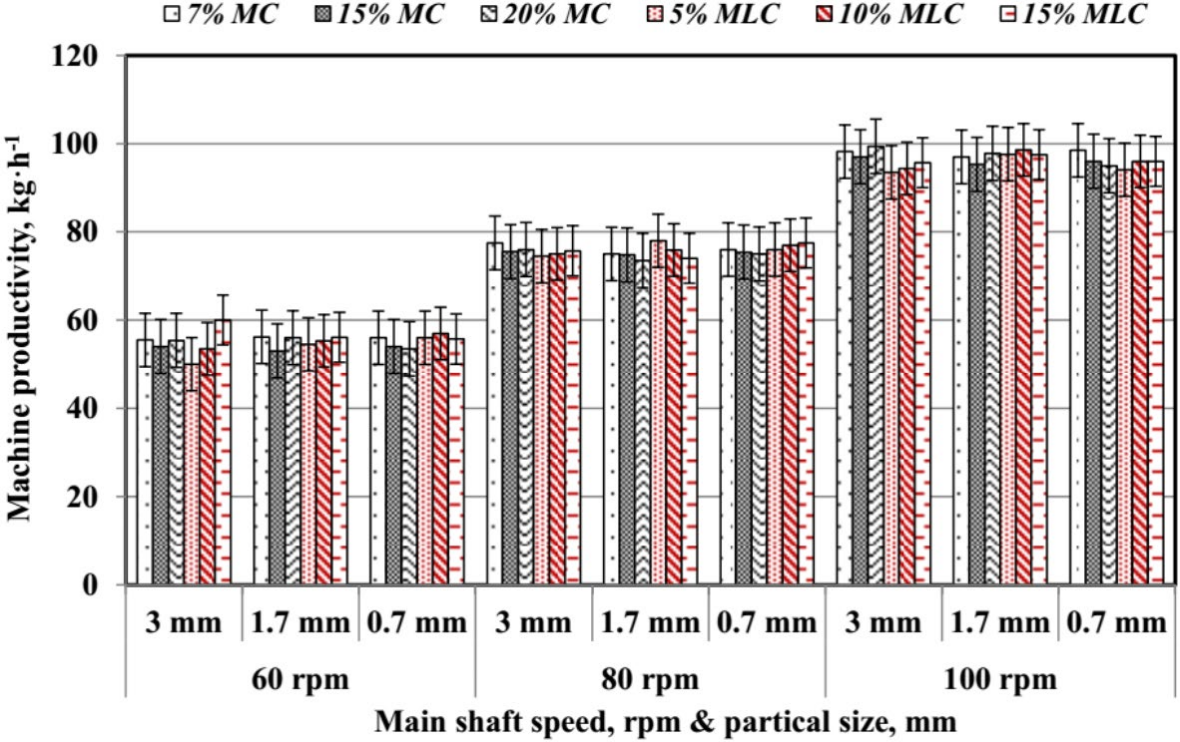
Machine productivity is influenced by speed, particle size, and additives.

The average values of *M*_*p*_ were illustrated in Fig. 17. The results revealed that *M*_*p*_ increased with higher moisture content (*MC*), molasses content (*MLC*), larger particle size (*PS*), and higher main shaft rotating speed (*RS*).

For 7% *MC*, *M*_*p*_ ranged from 55.5 kg h^−1^ at 3 mm *PS* and 60 rpm to a maximum of 98.5 kg h^−1^ at 0.7 mm *PS* and 100 rpm. For 15% *MC*, *M*_*p*_ reached 97 kg h^−1^ at 3 mm *PS* and 100 rpm. Similarly, for 20% *MC*, *M*_*p*_ peaked at 99.4 kg h^−1^ under the same conditions, emphasizing the role of higher moisture in improving machine productivity. Molasses content exhibited a more substantial impact. At 5% *MLC*, *M*_*p*_ ranged from 50 to 97.6 kg h^−1^, increasing with *PS* and *RS*. At 10% *MLC*, *M*_*p*_ reached 98.6 kg h^−1^ at 1.7 mm *PS* and 100 rpm, whereas 15% *MLC* resulted in a peak *M*_*p*_ of 97.5 kg h^−1^ at 1.7 mm *PS* and 100 rpm. These findings align with Sarker et al.^32^ and Nielsen et al.^33^, who reported that, increased *MC* and *MLC* enhance binding properties, leading to higher productivity. Optimizing *PS*, *RS*, and additive levels improves *M*_*p*_, demonstrating the combined effect of these factors in biomass pelletization processes. Considering these observations, the interactions among moisture content, molasses content, particle size, and rotating speed critically determine both pellet quality and machine throughput. Increasing *MC* and *MLC* improves the binding capacity of the raw material, resulting in more cohesive pellets and enhanced throughput, whereas larger particle sizes reduce resistance during pellet formation and higher *RS* improves mixing and compression. However, each parameter must be carefully balanced; excessive moisture can lead to downstream drying challenges and higher energy demands, while too much molasses may increase material costs and risk residue buildup. Similarly, although finer particles can improve binding, they may also increase friction and heat generation, potentially compromising pellet uniformity. Moreover, incorporating preconditioning steps such as adjusting the raw material temperature prior to pelletization can further stabilize the process by enhancing the interaction between moisture, molasses, and particles.

### Pellet quality

#### Pellet length (*Pl*)

**Fig. 18:**
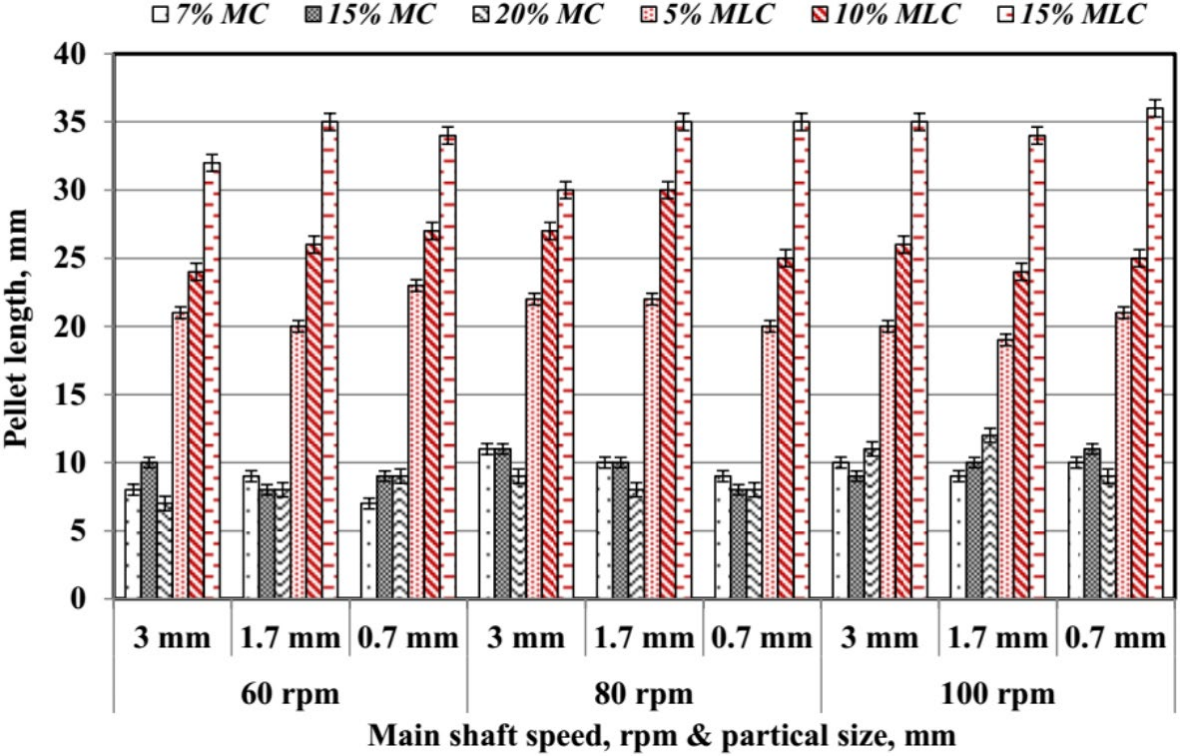
Pellet length at different speed, particle size and different additives.

The average values of pellet length (*Pl*) are illustrated in Fig. 18. *Pl* increased with higher molasses content (*MLC*), moisture content (*MC*), larger particle size (*PS*), and higher main shaft rotating speed (*RS*). For 7% *MC*, *Pl* ranged from 7 mm at 0.7 mm *PS* and 60 rpm to a maximum of 11 mm at 3 mm *PS* and 80 rpm. Similarly, for 15% *MC*, *Pl* ranged between 8 and 11 mm, while for 20% *MC*, it reached a maximum of 12 mm at 1.7 mm *PS* and 100 rpm. Molasses content significantly influenced *Pl*, especially at higher levels. At 5% *MLC*, *Pl* ranged between 19 and 23 mm, depending on *PS* and RS. At 10% *MLC*, *Pl* varied between 24 and 30 mm, while 15% *MLC* resulted in the highest values, reaching up to 36 mm at 0.7 mm *PS* and 100 rpm. These results demonstrate the role of *MLC* in enhancing pellet cohesion and length. The findings are consistent with Sarker et al.^32^, who highlighted the positive correlation between additive content and pellet quality. The data emphasize the importance of optimizing both *MC* and *MLC* alongside *PS* and *RS* to achieve the desired pellet length, crucial for improving the efficiency of pellet production processes.

#### Pellet particle density (***ρ***_***p***_)

**Fig. 19:**
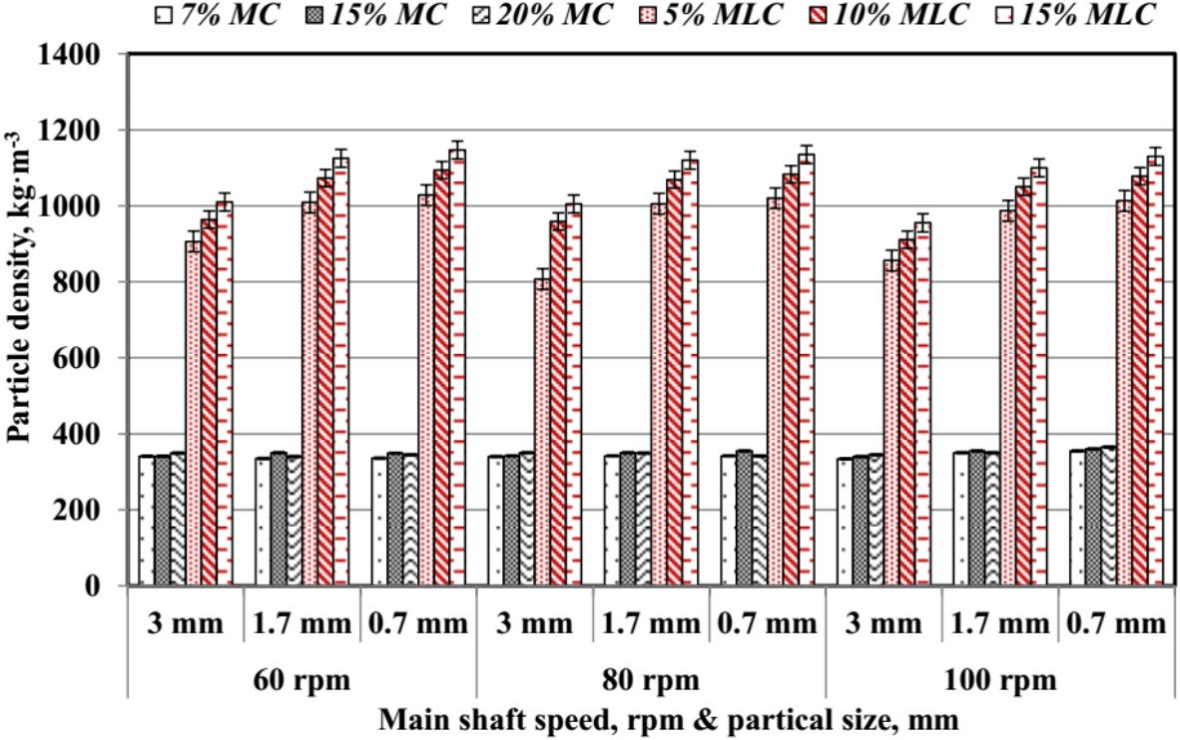
Pellet particle density at different speed, particle size, and additives.

The average values of pellet particle density (*ρ*_*p*_) are illustrated in Fig. 19. The main shaft rotating speed (*RS*), particle size (*PS*), and type of additive influence *ρ*_*p*_. Specifically, increasing *RS* from 60 to 100 rpm generally increased *ρ*_*p*_ for all treatments.

Additives with higher moisture content (*MC*) exhibited relatively stable *ρ*_*p*_ across different *RS* and *PS*, with values ranging from 335 to 365 kg m^−3^. This stability can be attributed to moisture’s role in enhancing inter-particle bonding, as supported by Pradhan et al.^34^. In contrast, additives containing molasses (*MLC*) produced significantly higher *ρ*_*p*_ values compared to *MC*-based additives. For example, 15% *MLC* yielded the highest density, reaching up to 1147 kg m^−3^ at 80 rpm and 0.7 mm *PS*, indicating that molasses enhances the binding and compaction properties of the pellets.

*MLC* treatments differed from *MC* treatments, with finer *PS* at higher *RS* contributing to improved *ρ*_*p*_ values. These results align with the findings of Nielsen et al.^33^ and Sarker et al.^32^, who reported that optimized pelletizing conditions, including higher rotational speeds and smaller particle sizes, lead to improved density and durability of biomass pellets.

#### Bulk density of the pellet (***ρ***_***b***_)

**Fig. 20:**
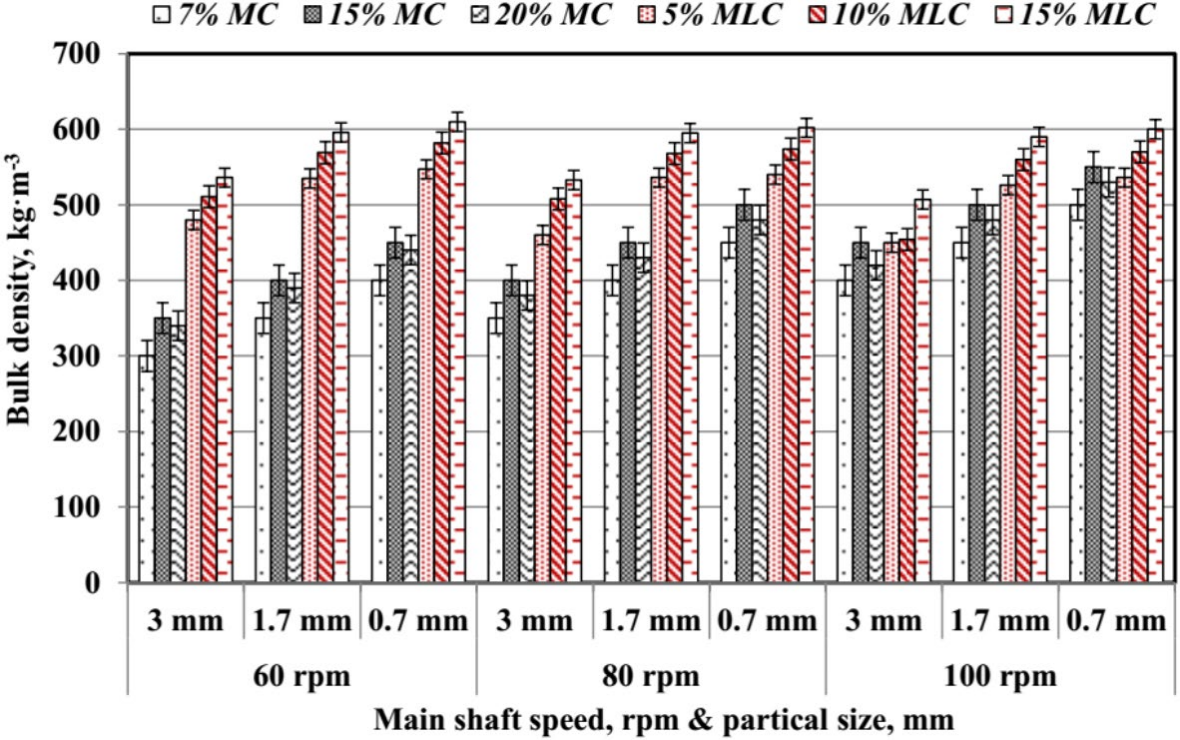
Bulk density of pellet at different levels of particle size, speed and additives.

The average values of pellet bulk density (*ρ*_*b*_) are shown in Fig. 20. The main shaft rotating speed (*RS*), particle size (*PS*), and type of additive have a pronounced impact on *ρ*_*b*_. Bulk density increased consistently with higher *RS* and smaller *PS* across all treatments. Additives with higher moisture content (*MC*) showed moderate increases in *ρ*_*b*_, ranging from 300 to 550 kg m^−3^, reflecting the contribution of moisture to compaction during pellet formation, as supported by Pradhan et al.^34^. Among molasses-based additives (*MLC*), higher values were observed.

For instance, 15% *MLC* produced the highest density, reaching up to 610 kg m^−3^ at 80 rpm and 0.7 mm *PS*, highlighting molasses’s superior binding properties, as confirmed by Nielsen et al.^33^. The 15% *MLC* achieved higher *ρ*_*b*_ compared to other treatments. These findings align with Nielsen et al.^33^, who demonstrated that optimal processing parameters enhance pellet properties, and Pradhan et al.^34^, who highlighted the importance of additives in improving biomass pellet density.

#### Pellet hardness resistance (*Hr*)

**Fig. 21:**
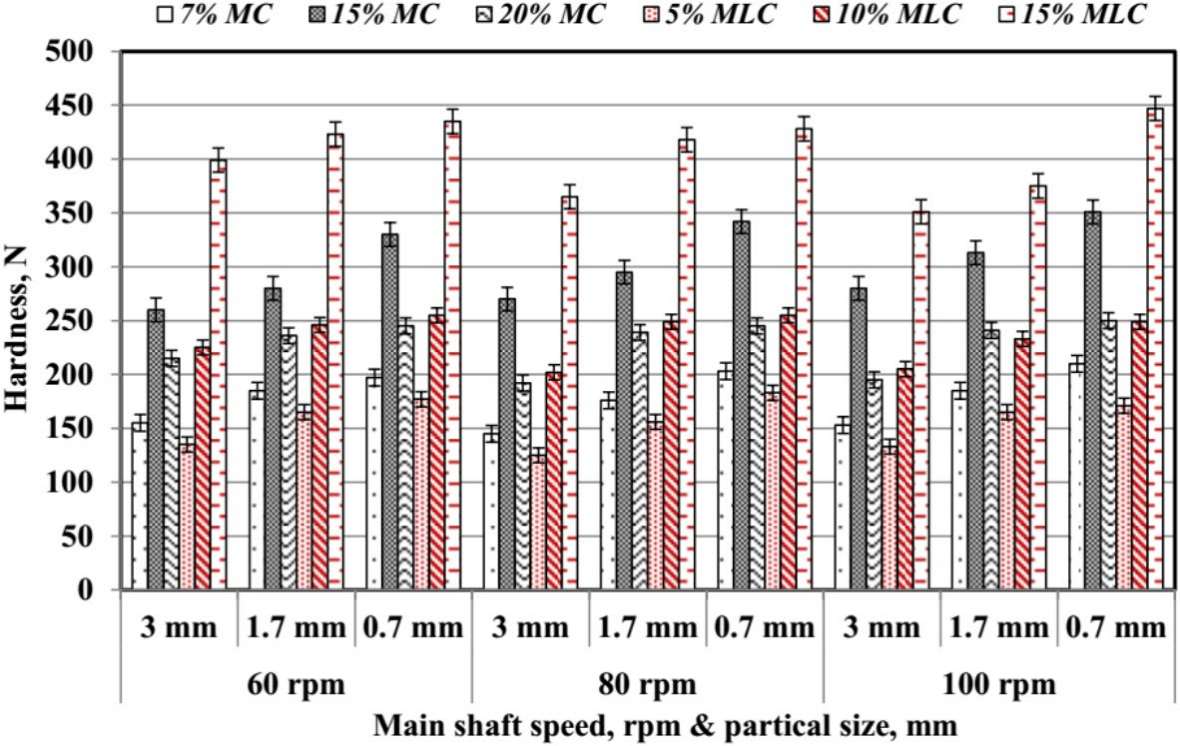
Hardness of pellet at different levels of particle size, speed and additives.

The average values of hardness resistance (*Hr*) are shown in Fig. 21. The main shaft rotating speed (*RS*), particle size (*PS*), and type of additive influence *Hr*, measured in Newtons. Higher *RS* and smaller *PS* generally led to increased *Hr* across all treatments. For moisture content (*MC*)-based additives, *Hr* ranged from 145 to 351 N, with 15% MC achieving the highest hardness, particularly at 100 rpm and 0.7 mm *PS*. This is consistent with findings from Pradhan et al.^34^, which highlighted the role of moisture in enhancing pellet durability. Molasses-based additives (*MLC*) exhibited significantly higher *Hr* values compared to *MC* treatments. For instance, 15% *MLC* achieved the highest *Hr* values, reaching 447 N at 100 rpm and 0.7 mm *PS*, underscoring the superior binding properties of molasses. The *RS*, *PS*, and additive type had effects on *Hr*. These results align with the findings of Nielsen et al.^33^, who emphasized the importance of mechanical and additive factors in optimizing pellet strength, and Sarker et al.^32^, who noted that molasses significantly improves the structural integrity of biomass pellets.

#### Shear stress of pellet (τ)

**Fig. 22:**
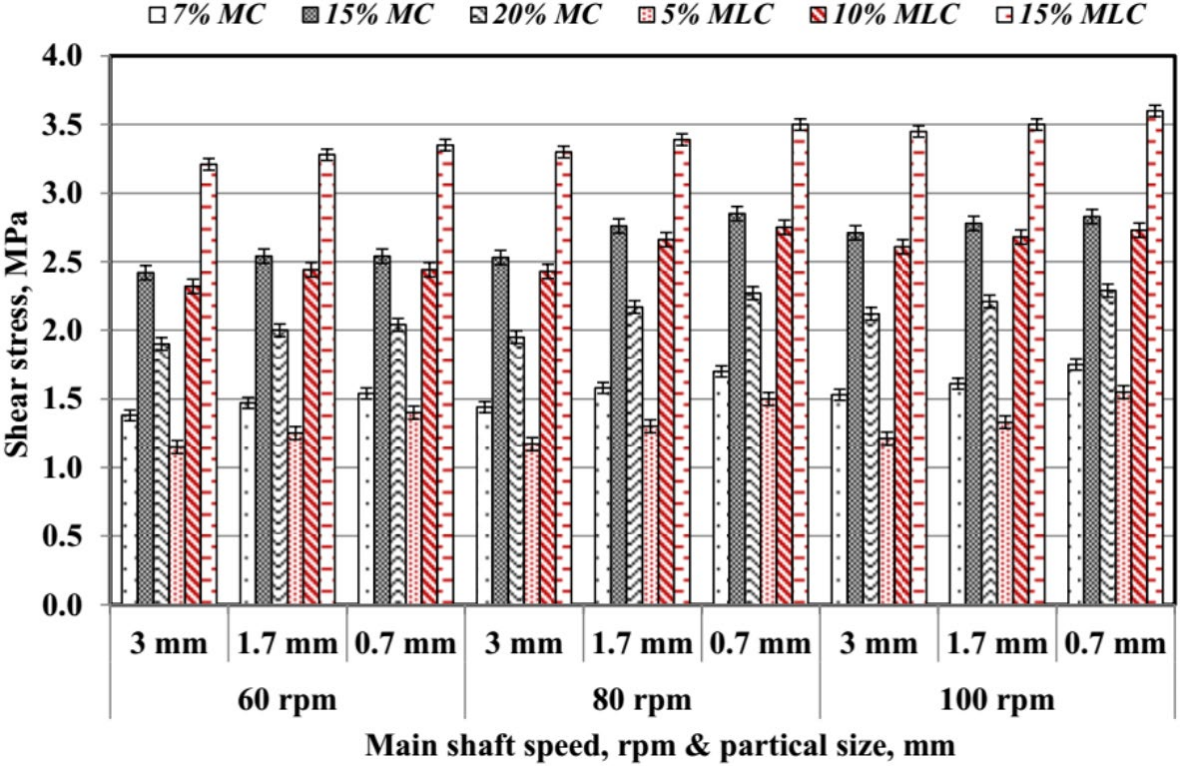
Shear stress of pellets by particle size, speed, additives.

The values of pellet shear stress (*τ*) are shown in Fig. 22. The *τ* increased with higher moisture content (*MC*) and molasses content (*MLC*). At 15% *MLC*, *τ* reached its maximum across all conditions, with values ranging between 3.21 and 3.6 MPa, compared to lower levels of additives, such as 7% *MC* or 5% *MLC*. This suggests that molasses content contributes more to shear stress than moisture content. For example, increasing moisture content from 7% *MC* to 15% *MC* showed a notable rise in shear stress at all *PS* and *RS*, with the highest difference observed at smaller particle sizes (e.g., 0.7 mm). Similarly, molasses at 15% showed higher values than 10% and 5% under comparable conditions, highlighting its superior binding capacity. The interaction between smaller *PS* and higher *RS* further amplified shear stress, likely due to improved compression and particle packing, as supported by earlier findings. Thus, molasses enhances binding strength more effectively than moisture, making it a crucial additive in pellet production.

#### Pellet durability (*Dp*)

**Fig. 23:**
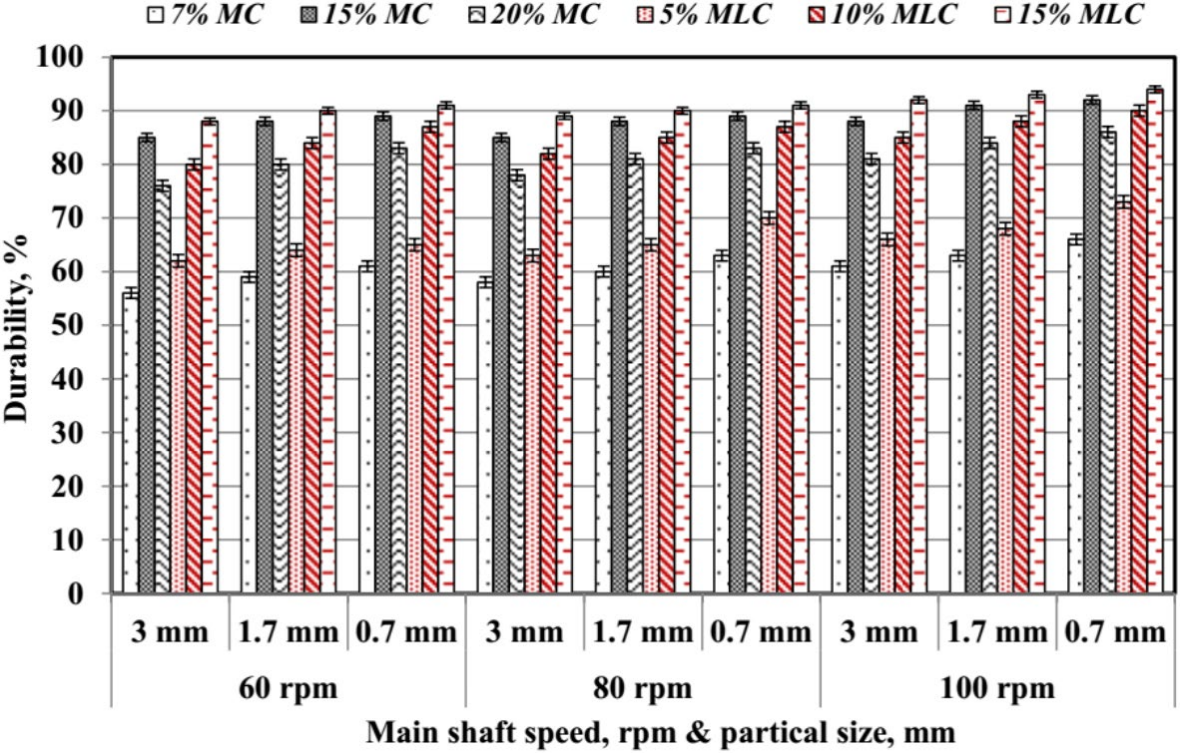
Pellet durability by speed, particle size, and molasses.

The average values of Pellet durability (*Dp*) are shown in Fig. 23. Durability increases with higher moisture content (*MC*) and molasses content (*MLC*). At 15% *MLC*, the durability values reached their peak, ranging from 88 to 94%, while the lowest values were observed at 7% *MC*, ranging between 56 and 66%. These results indicate that *MLC* has a stronger binding effect than *MC*, enhancing the *Dp* of pellets. Increasing *MC* from 7% *MC* to 15% *MC* led to a substantial rise in *Dp*. At 100 rpm with 0.7 mm particles, durability increased from 66% for 7% *MC* to 92% for 15% *MC*. Similarly, molasses improved durability; 15% *MLC* consistently outperformed 10% and 5% *MLC*, particularly at smaller particle sizes and higher shaft speeds, where enhanced compression and bonding were evident. The interaction between smaller particle sizes and higher *RS* further boosted *Dp*, likely due to better particle rearrangement and increased surface contact during compression. These findings align with the work of Ungureanu et al.^35^ and Mišljenović et al.^36^, who emphasized the importance of binders and particle size in improving pellet quality. Furthermore, Wang et al.^37^ and Whittaker and Shield^38^ highlighted the critical role of process parameters, such as speed and additives, in achieving high pellet durability. These results demonstrate that using molasses, particularly at 15%, is an effective strategy for enhancing pellet strength and durability, making it a valuable additive in pellet production systems.

Balancing machine productivity (*Mp*) and pellet durability (*Dp*) involves carefully adjusting moisture content (*MC*), molasses content (*MLC*), particle size (*PS*), and main shaft rotating speed (*RS*) to achieve both high throughput and robust pellet quality. As Figs. 17 and 23 indicate, higher *MC* and *MLC* can boost productivity and durability, yet excessive values risk process inefficiencies and potential buildup. Likewise, while smaller *PS* enhances pellet cohesion and durability, it may increase friction and reduce throughput. Conversely, larger PS eases material flow but can compromise mechanical strength. Therefore, adopting moderate *PS* and *RS* levels along with controlled *MC* and *MLC* can mitigate these trade-offs. Preconditioning steps, such as temperature adjustments, further stabilize the process, and real-time monitoring enables proactive parameter tuning. By systematically each factor in tandem, producers can establish operating conditions that sustain high production rates without undermining pellet integrity, ensuring a well-rounded balance between efficiency and quality.

#### Gross calorific value (GCV)﻿ and ash content

The gross calorific value (*GCV*) and ash content of ground cotton stalks were determined without any added *MC* or *MLC*, making the measured heating value equivalent to the higher heating value. The results revealed a gross heating value of 3938 kcal kg^−1^ and an ash content of 3.4%. These results indicate that cotton stalks have a relatively high heating value and a low ash content, making them a suitable biomass fuel source. The low ash content is particularly advantageous, as it minimizes the challenges associated with ash management during combustion. High ash content fuels can lead to issues such as the deposition of alkaline minerals, especially potash, on heat exchanger tubes. This deposition tends to occur due to the volatilization of these minerals during combustion, followed by condensation on surfaces, particularly in superheater regions. Additionally, the presence of alkaline minerals can lower the ash sintering temperature, resulting in slagging and fouling on boiler surfaces, which can reduce efficiency and increase maintenance costs^39^.

In general, *MC* significantly affects both pellet quality and process efficiency by influencing the plasticity and binding capacity of the raw material; too little moisture can lead to poor adhesion and low pellet durability, while excessive moisture may result in over-compaction and inconsistent density. *MLC* serves as an effective binder, enhancing particle cohesion and improving the mechanical properties of the pellets, such as hardness resistance and shear strength, as evidenced by the increased pellet lengths and densities observed with higher *MLC* levels. Particle size (*PS*) directly impacts the packing efficiency and surface contact between particles; a finer *PS* generally facilitates better compaction and uniformity, though overly fine particles can increase friction and potentially reduce throughput. Additionally, the main shaft rotating speed (*RS*) determines the force applied during the pelleting process, with higher speeds promoting greater compressive forces that enhance pellet density and durability, yet excessively high speeds may induce machine wear or cause energy inefficiencies.

#### Comparison between standard and obtained experimental pellet quality criteria

Comparison between the standard pellet quality criteria and the obtained experimental values reveals several critical insights. According to DIN 51731^40^ and ENplus guidelines^41^, high-quality pellets should exhibit a length (*Pl*) of 20–30 mm, a pellet particle density (*ρ*_*p*_) of 1000–1400 kg m^−3^, a bulk density (*ρ*_*b*_) of 600–800 kg m^−3^, hardness resistance (*Hr*) between 300 and 500 N, shear stress (*τ*) around 3–4 MPa, pellet durability (*Dp*) exceeding 90% (typically reaching 94–95%), a gross calorific value (*GCV*) of 16–18 MJ kg^−1^, ash content below 6–8%. In contrast, experimental results demonstrated that pellet length is highly dependent on processing conditions and additives; treatments with 7% moisture content (*MC*) produced lengths of 7–12 mm, which fall below the standard range, whereas the use of molasses-based additives (*MLC*) at 10–15% yielded lengths ranging from 24 to 36 mm, with some values slightly exceeding the upper standard limit. Furthermore, while *MC*-based pellets showed pellet particle densities of only 335–365 kg m^−3^, the optimized 15% *MLC* treatment achieved a density of up to 1147 kg m^−3^, well within the desired range. Bulk density for 15% *MLC* reached 610 kg m^−3^, aligning closely with standard expectations, whereas *MC* treatments lagged. Hardness resistance, shear stress, and durability values recorded for pellets produced with 15% *MLC*—447 N, 3.21–3.6 MPa, and up to 94%, respectively further corroborate their superior quality, as supported by Nielsen et al.^33^ and Sarker et al.^32^. Additionally, the measured *GCV* of approximately 16.46 MJ kg^−1^ and an ash content of 3.4% are consistent with standards. These comparisons suggest that the incorporation of molasses at around 15%, alongside optimized moisture content, particle size, and rotational speed, is essential to produce pellets that not only meet but potentially exceed international quality standards, thereby offering a sound recommendation for industrial pellet production.

### Energy consumption of pelleting process

Table 2 summarizes the energy consumption during the two main stages of the pelleting process: chopping, grinding and pelleting. Ancillary processes such as collection and transportation are not included in the table. The energy consumption for chopping and grinding was calculated as 23.22 kW h ton^−1^ of residue. The labor energy was calculated where the chopping and grinding process requires two laborers. So, the labor energy for chopping and grinding is calculated as 0.08 kW h ton^−1^ of residue. For the pelleting process, the energy requirement of the pelleting machine was previously calculated at a machine speed of 100 rpm, resulting in an energy consumption of 103.60 kW h ton^−1^ of residue. The pelleting process also requires two laborers, and the machine productivity is 96 kg h^−1^. The labor energy for pelleting is calculated as 1.55 kW h ton^−1^ of residue. The total energy consumption for the pelleting process, including chopping, grinding and labor energy, is therefore the sum of all the calculated energy components, amounting to 128.45 kW h ton^−1^ of residue. Given that the heating value of cotton stalks is 3938 kcal kg^−1^, it can be observed that the energy consumed during the pelleting process represents only about 2.8% of the total energy potential of the cotton stalks. This demonstrates that the pelleting process is energy-efficient compared to the energy output of the cotton stalks.

**Table 2:** Energy consumption of the pelleting processes compared with *GCV*.

Process	Mechanical energy (kW h ton^−1^)	Labor energy (kW h ton^−1^)
Chopping and grinding	23.22	0.08
Pelleting	103.60	1.55
Sum	126.82	1.63
Total Energy, (kW h ton^−1^)	128.45	
*GCV* of cotton stalks, (kW h ton^−1^)	4576.9	

### Heatmap analysis

**Fig. 24:**
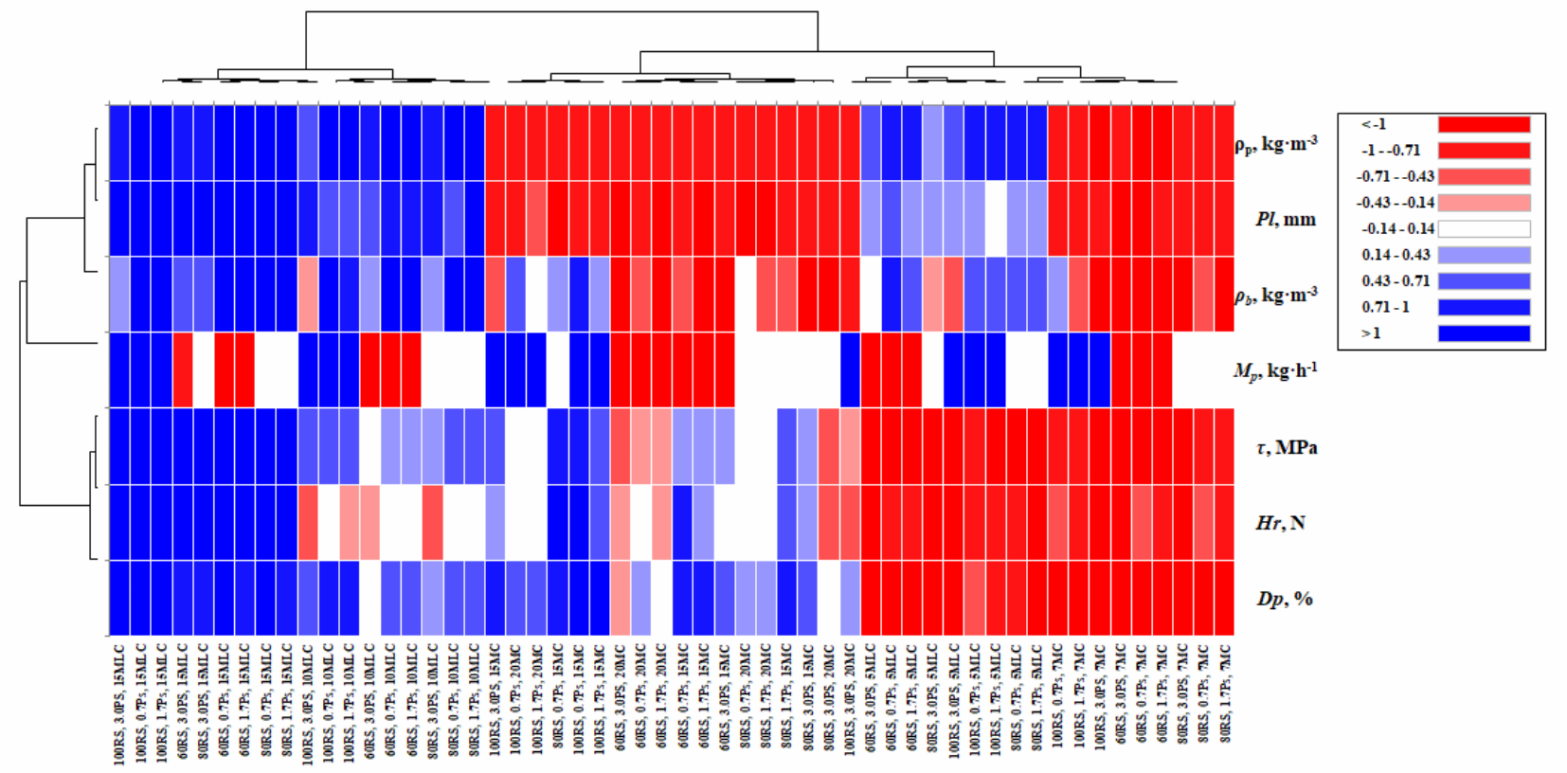
Heatmap of evaluation criteria for pelleting machine performance.

The heatmap (Fig. 24) illustrates the correlations among various parameters influencing pellet quality, including machine productivity (*M*_*p*_), pellet length (*Pl*), particle density (*ρ*_*p*_), bulk density (*ρ*_*b*_), hardness resistance (*Hr*), shear stress (*τ*), and durability (*Dp*). It is evident that molasses content (*MLC*) significantly improves pellet properties, as indicated by strong positive correlations (blue tones) between *MLC* levels and *Dp*, *Hr*, and *τ*. At 15% *MLC*, durability and hardness reached their highest values, reflecting enhanced binding and structural integrity.

All variables were normalized using *z-score* standardization (subtracting the mean and dividing by the standard deviation) to ensure comparable scales and prevent any single parameter from dominating the analysis. The Pearson correlation coefficient was used to measure the linear relationships, where coefficients range from − 1 (strong negative) to + 1 (strong positive), and 0 indicates no linear relationship; in the heatmap, red tones represent negative correlations while blue tones indicate positive ones. The results show that molasses content (*MLC*) has strong positive correlations with *Dp*, *Hr*, and *τ*, suggesting that increasing *MLC* significantly enhances pellet properties, as evidenced by the highest values of durability and hardness at 15% *MLC*. Moisture content (*MC*) also exhibits moderate positive correlations with *τ* and *Dp*, although its impact is less pronounced compared to *MLC*. Additionally, higher rotating speeds (*RS*) and smaller particle sizes (*PS*) lead to improved compaction and densification, thus increasing *ρ*_*p*_ and *ρ*_*b*_, which aligns with the findings of Jiang et al.^42^. Productivity (*Mp*), however, shows a slight trade-off with durability and hardness, especially at higher speeds and smaller particle sizes, indicating that a balance between efficiency and pellet quality is necessary. Negative correlations between pellet length (*Pl*) and both *τ* and *Dp* suggest that longer pellets may have reduced structural strength due to uneven compaction. These observations underscore the importance of optimizing *MLC*, *MC*, *RS*, and *PS* to achieve superior pellet properties, a conclusion that agrees with Ungureanu et al.^35^, who also reported the synergistic effects of process parameters and binding additives on enhancing pellet performance.

Overall, the evaluation of machine performance highlights the effectiveness of the pelleting process in achieving high productivity while maintaining pellet quality. The results demonstrate that optimizing moisture content (*MC*), molasses content (*MLC*), particle size (*PS*), and main shaft rotating speed (*RS*) is essential for balancing throughput and mechanical strength. The machine operated efficiently, with a total energy consumption of 128.45 kW h ton^−1^, representing only 2.8% of the total energy potential of the produced pellets, confirming its energy efficiency. Furthermore, the highest durability (94%) and density (610 kg m^−3^) were achieved at optimized settings, ensuring superior pellet integrity. These findings underscore the importance of precise parameter control in maximizing both machine efficiency and pellet quality.

## Conclusions

This study highlights the existing research gap in integrating advanced numerical simulation methods, such as the Finite Element Method (*FEM*), with practical pelleting machine design. While significant advancements have been made in improving pelleting parameters, the need for further optimization integrating these methods with practical machine performance remains. This study demonstrated that enhancing pelleting process parameters significantly enhances both pellet quality and production efficiency. Key findings indicate that machine productivity (*Mp*) increased with higher moisture content (*MC*), molasses content (*MLC*), larger particle size (*PS*), and higher main shaft rotating speed (*RS*), reaching peak values at 15% *MLC* and 100 rpm. The results further revealed that pellet length (*Pl*) and durability (*Dp*) improved notably with increased *MLC* and *MC*, highlighting the superior binding and cohesive properties of molasses, particularly at 15% *MLC*. Pellet particle density (*ρ*_*p*_) and bulk density (*ρ*_*b*_) were strongly influenced by *RS*, *PS*, and additive content. Molasses-based pellets achieved significantly higher densities compared to moisture-based ones, emphasizing molasses’ critical role in enhancing pellet compaction. Additionally, hardness resistance (*Hr*) and shear stress (*τ*) exhibited maximum values with finer *PS*, higher *RS*, and 15% *MLC*, further reinforcing the importance of molasses in improving structural integrity. The study also confirmed that durability increased with higher *MLC* and *MC*, reaching peak values of 94% at 15% *MLC*, underscoring molasses’ superior contribution to pellet cohesion. Energy consumption analysis revealed that the pelleting process was highly efficient, requiring only 2.8% of the total energy potential of cotton stalks. This highlights the viability of cotton stalks as a sustainable biomass fuel source with relatively low ash content (3.4%), which minimizes combustion-related issues such as slagging and fouling. The study also emphasized the trade-offs in optimizing pelleting parameters; while increased *MC* and *MLC* enhance pellet cohesion and durability, excessive levels may introduce drying challenges and additional material costs. Similarly, finer *PS* improves binding but may lead to higher friction and heat generation, potentially compromising pellet uniformity.

## Electronic supplementary material

Below is the link to the electronic supplementary material.

Supplementary Material 1

## Data Availability

The datasets generated and/or analyzed of the current study are available from the corresponding author on reasonable request.

## Abbreviations

*ω*: Angular velocity of the main shaft = $\frac{2\pi n}{{60}}$, rad s^−^^1^
*µ*: Friction coefficient
*A*: Area of the pellet, mm^2^
*Ai*: Inlet area of orifice before extrusion, mm^2^
*Ao*: Outlet area of orifice after extrusion, mm^2^
*A*_*S*_: The orifice surface area = $\pi d_{o} L$, mm^2^
*CE*: Energy consumption, kW h ton^−^^1^
*d.b*: Dray bases
*D*_1_: Diameter of the motor pulley, mm
*D*_2_: Diameter of the machine pulley, mm
*Di*: Inlet diameter of orifice before extrusion, mm
*Do*: Outlet diameter of orifice after extrusion, mm
*Dp*: Pellet durability, %
*D*_*P*_, *D*_*g*_: Pitch cycle diameter of pinion and gear, respectively, mm
*D*_*roller*_: Distance between rollers, mm
*E*_1_: Young’s modulus of the cylinder, N m^−^^2^
*E*_2_: Young’s modulus of the plane, N m^−^^2^
*EL*: Labor energy, kW h ton^−^^1^
*F*_1__*-roller*_: Total force of the pelleting process for one roller, N
*F*_*die*_: Friction force due to the moving the pellet through hole, N
*FEA*: Finite Element Analysis
*F*_*ext*_: Extrusion force of ground material into the orifice, N
*F*_*roll*_: Rolling force, N
*GCV*: Gross calorific value, kW h ton^−^^1^
*h*_1_: Height of the ground material in front of the roller, mm
*h*_2_: Height of the ground material after of the roller, mm
*Hr*: Hardness resistance, N
*ID*: Inside diameter, mm
*L*: Roller length, mm
*L*_*h*_: Length of the straight section of the hole, mm
*L*_*p*_: Average power of the labor, HP
*L*_*s*_: Distance from the roller to the head, mm
*M*_*A*_: Mass of pellet after deformation, kg
*M*_*b*_: Bending moment, N m
*M*_*B*_: Mass of pellet before deformation, kg
*MC*: Moisture content, %
*MLC*: Molasses content, %
*Mp*: Machine productivity, kg h^−^^1^
*N*: Number of orifices that roller covers them simultaneously
*n*: Rotational speed of the main shaft, rpm
*n*_1_: Rotational speed of the electric motor, rpm
*n*_2_: Rotational speed of transmission shaft, rpm
*n*_3_: Rotational speed of the main shaft, rpm
*N*_*L*_: Number of labor
*n*_*P*_, *n*_*g*_: Rotational speed of pinion and gear, respectively, rpm
*N*_*P*_, *N*_*g*_: Number of teeth of pinion and gear, respectively
*NREA*: New and Renewable Energy Authority
*O*_*ar*_: Orifice area ratio $= \frac{{A_{i} }}{{A_{o} }} = \frac{{d_{i}^{2} }}{{d_{o}^{2} }}$
*P*_*ext*_: Extrusion pressure $= \sigma_{y} \ln O_{ar}$, N mm^−^^2^
*Pl*: Pellet length, mm
*PS*: Particle size, mm
*Ptd*: Track of roller = $\frac{\pi }{2}D$_die_ (for two roller), mm
*R*: Roller radius, mm
*RP*: Required power, W
*rpm*: Revaluation per minute
*RS*: Main shaft rotating speed, rpm
*S*_2_: Number of teeth in pinion
*S*_3_: Number of teeth in gear
*T*: Torque that applied on the main shaft, N m
*W*: Total force acting the gear teeth, N
*W*_*a*_: Axial force, N
*W*_*r*_: Radial force, N
*W*_*t*_: Tangential force, N
*w*: Roller width, mm
*γ*_1_: Poisson’s ratio of the roll (cylinder)
*γ*_2_: Poisson’s ratio of the plane
*Δh*: *h*_2_ − *h*_1_, mm
*μ*_*roll*_: Coefficient of friction (rolling) between the ground material & die
*ρb*: Bulk density, kg m^−^^3^
*ρp*: Particle density, kg m^−^^3^
*σ*_*f*__1_: Mean flow stress of the ground material before rolling, N mm^−^^2^
*σ*_*f*__2_: Mean flow stress of the ground material after rolling, N mm^−^^2^
*σ*_*fm*_: Mean flow stress of the ground material, N mm^−^^2^
*τ*: Shear stress, MPa
