# Supplementary material for: Design and performance assessment of a pelleting machine for sustainable biomass pellet fuel production from plant residues
Source: Sci Rep. 2025 Apr 15;15:12879. doi: 10.1038/s41598-025-93058-6 (PMC12000602; doi:10.1038/s41598-025-93058-6)
Supplement: Supplementary file 1 — Supplementary Material 1 [file 41598_2025_93058_MOESM1_ESM.docx]

**Supplementary Material**

**S.1 Material preparation**

**S.1.1 Cotton stalks characteristics**

Cotton stalks (‎*Gossypium hirsitum L.*, variety Giza 90) were obtained from the farm of Faculty of Agriculture, Cairo University, Giza, Egypt. The physical properties of stalks are shown in Table S1.The dimensional characteristics of cotton stalks were 145 cm mean length, 13.2 mm mean diameter.

**Table S1. Physical properties of cotton stalk.**

| Stem diameter (mm) | | Stem length (mm) | Moisture content (d.b), % | Bulk density, $\rho$ (kg·m^-3^) |
| --- | --- | --- | --- | --- |
| Bottom | Top |  |  |  |
| 8-16 | 4-8 | 710-1850 | 7 | 80 |

**S.1.2 Chopping the cotton stalks**

Cutting of cotton stalks can be performed by many types of chopping machines. The cutting machine, which was used in this work, is illustrated in Figure S1, where the cutter-head of this machine is from the type of flywheel drum. In this machine, the cutter has two radial knifes, which are fastened on it by three bolts and rotate with high speed corresponding to lower speed of feeding drum. The feeding drum in this machine is an assistant element in the cutting process, which was used to push the plant stems to the cutter-head for cutting. After chopping, the chopped stalks were tested for the size in Central Laboratory in Agricultural Engineering Research Institute- Ministry of Agriculture. The chopping machine specifications as the following:

1) General features: - Vermeer manufacturing company, U.S.A, self-Propelling Machine

‎- Overall length: 267 cm ‎- Overall width: 122 cm ‎- Overall height: 226 cm ‎- Total weight: 780 kg - Cutter-head diameter: 60 cm ‎‎- Power transmission type: “V” shape belts‎

- Knife dimensions: 200 mm length, 80 mm height and 16 mm flank thickness.

2) Chopping capacity: Maximum stem diameter: 15.2 cm

‎3) Engine: - Model: Honda GX620‎ ‎- Max. Power: 20 HP (14.9 kW) at 3600 rpm.‎

‎- Engine oil capacity: 15 liters with filter.‎ ‎- Fuel tank capacity: 27 liters ‎- Fuel type: Benzine.‎ ‎- Cooling medium: air.‎

4) Hydraulic system: - Pump flow rate: 22.3 liters·min^-1^. - System pressure: 172 bar maximum pressure. - Oil reservoir capacity: 17 liters.


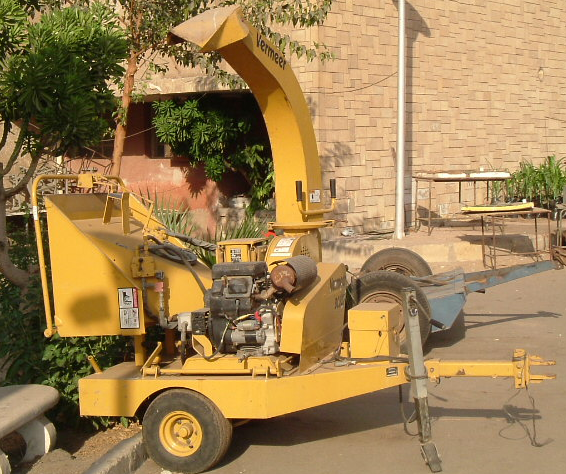


**Figure S1. Chopping machine.**

**S.1.3 Grinding the cotton stalks**

The hammer mill (WILEY CUTTING MILL, Type 1029-B- YOSHIDA SEISAKUSHO CO. LTD, TOKYO-JAPAN) was used for grinding biomaterials, as shown in Figure S2. Hammer mills reduce particle size of solid materials using both shear and impact action. The hammer mill used in this study consisted of 8 hammers, attached to a shaft powered by a 0.4 kW electric motor (220 V). The shaft rotated at a speed of 990 rev·min^-1^. The material was crushed or shattered by the repeated hammer impacts, collisions with the walls of the grinding chamber, as well as particle–on–particle impacts. Perforated metal screens covering the discharge opening of the mill retained coarse materials for further grinding while allowing the properly sized materials to pass as finished product. This machine was used for grind the material to study the compaction behavior of the material.


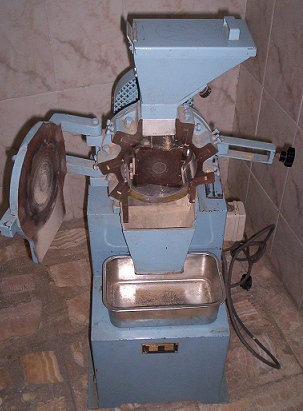


**Figure S2. The grinding machine**.

**S.1.4 Particle size distribution**

A sample grind of 100 g was placed in a stack of sieves arranged from the largest to the smallest opening. The sieve series selection was based on the range of particles in the sample. For grinds material, sieve numbers were 2, 1.4 and 0.7. Sieve analysis was repeated three times for each ground samples. The particle size was determined according to ANSI/ASAE standard S319.3JUL97 (Standard, 2002). Typical distribution of the mass percentage of cotton stalks grinds retained on individual test sieves is shown in Table S2‎.

**Table S2.‎ Particle size distribution for grinding cotton stalks.**

| Particle size, (mm) | ≤ 0.7 | 0.7-1.4 | 1.4-2 | 2-3 |
| --- | --- | --- | --- | --- |
| Percentage of Particle size, (%) | 45.5 | 39.0 | 7.5 | 8.0 |

**S.2 Design of the different parts of the pelleting machine**

A sketch of the pelleting machine and its main components are presented in Figure S3.

**
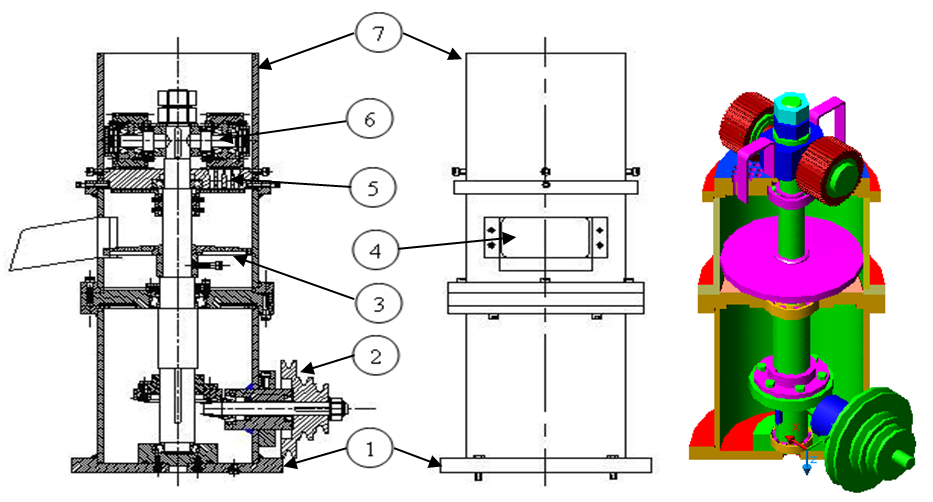
**

**Figure S3.** Schematic diagram of the pelleting machine: 1. Mounted base, 2. Transmission unit, 3. Collected tray, 4. Hopper, 5. Die, 6. Pelleted rollers, 7. Input orifice

**‎S.2‎.1 Design of the pelleting shaft**

The unit that carries the roller consists of two shafts and head that connected with the main shaft. Each shaft is fitted to the head with a translation fit, Figure S4‎.


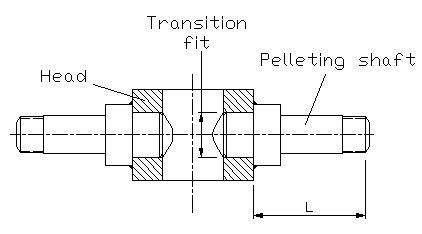


**Figure S4.** The Pelleting shafts and the head.

Therefore, Shaft was designed through the following procedures. Maximum bending moment applying on shaft is Figure S5‎:

| $M_{b}\text{ = }F_{1-roller}\text{ . Ls}$ | | | **(S1)** |
| --- | --- | --- | --- |
| Where; | |  | |
| M_b_ | **:** | Bending moment. | |
| Ls | **:** | Distance from the roller to the head, as shown in Figure S9. | |

The ASME code equation for shafts subjected to torsion, bending, and axial loads by applying the maximum shear equation modified by introducing, shock, fatigue and column factors as follows:

**M_max_= 102567 N‎**·**‎mm**

**50 mm**

**F_1-roll_ = ‎2051.34‎ N**

**R_1_= ‎2051.34‎ N**

**B. M. D.**

**Figure S5.** Pelleting shaft bending moment.

| $\text{d}^{3}\text{=}\frac{16}{\text{πS}_{\text{s}}\text{(1-K}^{4}\text{)}}\sqrt{\left[\text{K}_{6}\text{M}_{6}\text{+}\frac{\text{αF}_{\text{a}}\text{d}_{0}\text{(1+K}^{2}\text{)}}{8}\right]^{2}\text{+}\left(\text{K}_{\text{t}}\text{M}_{\text{t}}\right)^{2}}$ | | | **(S2)** |
| --- | --- | --- | --- |
| Where; | |  | |
| M_t_ | **:** | Torsional moment, N·m | |
| M_b_ | **:** | Bending moment, N·m | |
| d_o_ | **:** | Shaft outside diameter, m | |
| d_i_ | **:** | Shaft inside diameter, m | |
| F_a_ | **:** | Axial load, N | |
| K | **:** | K = $\frac{d_{i}}{d_{o}}$ | |
| K_b_ | **:** | Combined shock and fatigue applied to bending moment. | |
| K_t_ | **:** | Combined shock and fatigue applied to torsional moment. | |
| S_s_ | **:** | Shear stress allowable. | |
| __ | **:** | Column action factor. | |
|  |  | $\propto=\frac{1}{1-0.0044(\frac{L}{\chi})}$ for L/$\chi$ >115 $\alpha=\frac{S_{y}}{\pi^{2}nE}(\frac{L}{\chi})^{2}$  for L/$\chi$ <115 | |
| n | **:** | 1,2.25,1.6 for hinged end, fixed, ends partly, | |
| $\chi$ | **:** | Radius of gyration = $\sqrt{I/A}$ | |
| I | **:** | Rectangular moment of inertia | |
| A | **:** | Crosse section area of shaft | |
| S_y_ | **:** | Yield stress in compression. | |
| $\text{K=}\frac{\text{d}_{\text{i}}}{\text{d}_{0}}\text{=}\frac{0}{\text{d}_{0}}\text{=0}$ | | | |
| K_b_ = 1.5 to 2.0 load suddenly applied (stationary shaft) | | | |
| S_allowable_ = Allowable shear strength of shaft value = 40 MP_a_. | | | |
| $\alpha$= 0 | | | |

The above equation gives; **d = 29.7 mm.** Therefore, diameter of pelleting shaft must be equal or more than **30** mm for design safety.

**S.2‎.2 Design of the die**

The die design was not easy. Geometry, contact and friction between fibrous materials and the die steel have to be considered. The problem is the complex mechanical behavior of the biological material to be processed. One option for designing the die is to manufacture different dies just by changing the angle of the tapered dies, length, and roughness.

**S.2‎.2.1 Die design**

The roll press are on the die is represented by using Hertzian equation, as shown in Figure S6‎. The case that represents roll press on the die is a cylinder on a plane. The local pressure on the contact area due to an applied load F is (Abdelbary & Chang, 2023):

| $\text{P=}\frac{\text{2F}}{\text{πaL}}\left(\text{1-}\frac{\text{x}^{2}}{\text{a}^{2}}\right)^{\frac{1}{2}}$ | | | **(S3)** |
| --- | --- | --- | --- |
| Where; | |  | |
| P | **:** | Local pressure in the contact area. | |
| F | **:** | Applied force. | |
| a | **:** | Half contact width. | |
| L | **:** | Length of the roller. | |
| x | **:** | General position, as shown in Figure S10. | |


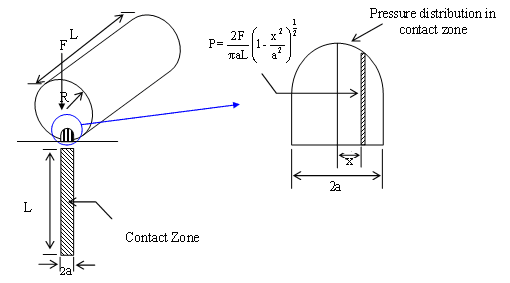


**Figure S6.** Representation of the contact stress (Hertzain stress) of roller on the die plat.

**S.2‎.2.2 Maximum contact stress (P_max_)**

The maximum pressure between the roller and the die can be calculated from the following equation, (Abdelbary & Chang, 2023):

| $\text{P}_{\text{max}}\text{=}\frac{\text{2F}}{\text{πaL}}$ | | | **(S4)** |
| --- | --- | --- | --- |
| $\text{a=}\left[\frac{\text{4FR}}{\text{πL}}\left(\frac{\text{1-γ}_{1}^{2}}{\text{E}_{1}}\text{+}\frac{\text{1-γ}_{2}^{2}}{\text{E}_{2}}\right)\right]^{\frac{1}{2}}$ | | | |
| Where; | | | |
| R | **:** | The radius of the cylinder. | |
| $\gamma_{1}$ | **:** | Poisson's ratio of the roll (cylinder). | |
| $\gamma_{2}$ | **:** | Poisson's ratio of the plane $\gamma$ = 0.3. | |
| E_1_ | **:** | Young's modulus of the cylinder. | |
| E_2_ | **:** | Young's modulus of the plane =200 $\times$ 10^9^ N·m^-2^. | |
| a = 0.15 mm | | | |
| P_max_ = 145.18 MPa | | | |

The annular distribution of the holes on the die as shown in the Figure S7‎ is 43, 37, 31, and 25 holes from the outer to inner diameter, respectively. The outer diameter of die and its thickness are 274 mm, 40 mm, respectively. The ratio between the opening area and the all area of the die is 0.367.

**
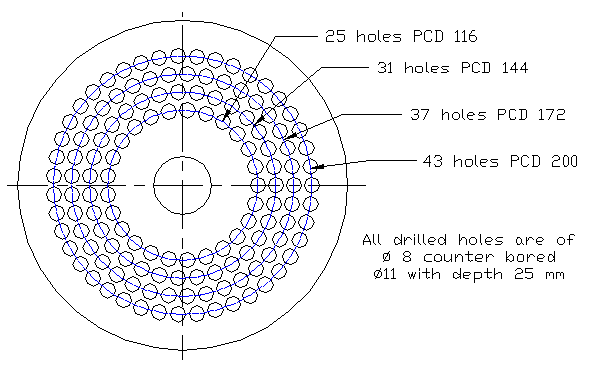
**

**Figure S7.** The distribution of the hole in the die.

**S.2‎.3 Design of the bevel gear**

Bevel gear is used to transmit the power from the transmission unit to the main shaft. The type of this gear is hypoid bevel gear.

**S.2‎.3.1 Gear kinematics**

The relation between the speeds of the two meshed gears is inverse proportional to the ratio of gear diameter and number of teeth, as the following:

| $\frac{\text{n}_{\text{P}}}{\text{n}_{\text{g}}}\text{=}\frac{\text{N}_{\text{g}}}{\text{N}_{\text{P}}}\text{=}\frac{\text{D}_{\text{g}}}{\text{D}_{\text{P}}}$ | | | **(S5)** |
| --- | --- | --- | --- |
| Where; | |  | |
| n_P_ , n_g_ | **:** | Rotational speed of pinion and gear, respectively. | |
| N_P ,_ N_g_ | **:** | Number of teeth of pinion and gear, respectively. | |
| D_P_, D_g_ | **:** | Pitch cycle diameter of pinion and gear, respectively. | |

**S.2‎.3.2 Force analysis**

Figure S8 shows the force analysis of the gear and pinion. The forces acting on teeth (W) of the two gears can be analysis into three components tangential, radial and axial forces as follows:

| $\text{W}_{\text{t}}\text{=H/}\omega\text{ r}_{\text{m}}$ $\text{W}_{\text{r}}\text{=W}_{\text{t}}\text{tanφcosγ}$ $\text{W}_{\text{a}}\text{=W}_{\text{t}}\text{tanφsinγ}$ $\text{W=}\left[\text{W}_{\text{t}}^{2}+\text{W}_{\text{a}}^{2}+\text{W}_{\text{r}}^{2}\right]^{1/2}$     | | | **(S6)** |
| --- | --- | --- | --- |
| Where; | |  | |
| W | **:** | Total force. | |
| W_t_ | **:** | Tangential force component. | |
| W_r_ | **:** | Radial force component. | |
| W_a_ | **:** | Axial force component. | |
| H | **:** | Transmitted power. | |
| r_m_ | **:** | Mean radius of pinion =$\frac{d_{P}}{2}-(\frac{F}{2})\sin\gamma$. | |
| $\gamma$ | **:** | Pinion pitch cone angle$\tan^{-1}(\frac{N_{P}}{N_{g}})$. | |
| d_P_ | **:** | Pinion pitch circle diameter. | |
| F | **:** | Face width. | |
| $\emptyset$ | **:** | Pressure angle. | |

**Figure S8.** Force analysis of the bevel gear and pinion.

For the gears used in suggested machine, bevel and pinion gears are chosen with the following data:

Pressure angle ($\varphi$) = 20^o^

Number of teeth in the pinion (N_P_) = 6

Number of teeth in the gear (N_G_) = 41

Diameter of gear (D_o_) = 150 mm

Diameter of pinion (do) = 35 mm

Pinion pitch cone angle $\left[\text{γ}\right]\text{=8.32}^{\text{o}}$

Face width f pinion (F) =30 mm

Rotational speed of pinion (n_P_) = 1572 rpm

Rotational speed of gear (n_g_) = 230 rpm

Power transmitted (H) = 7500 W

$$\text{r}_{\text{m}}\text{ [pinion] =}\frac{35}{2}\text{-}\left(\frac{30}{2}\right)\text{sin8.32=15.33mm}$$

$$\text{r}_{\text{m}}\text{ [Gear] =}\frac{150}{2}\text{-}\left(\frac{30}{2}\right)\text{sin8.32=72.8 mm}$$

Ꞷ = 164.5 rad/sec

W_t_ = 2974.1 N

W_r_ = ‎2974.1‎×tan20 × cos 8.32 = 1071.1 N

W_a_ = ‎2974.1‎×tan20 × sin 8.32 = 156.6 N

**S.2‎.3.3 Design of bevel gear based on strength consideration**

The actual stress that acts on gear was calculated by applying ***Lewis*** equation, **(**Uicker et al., 2023**)**:

|  $\sigma\text{=}\frac{\text{2M}_{\text{t}}}{\text{m}^{2}\text{bπYN}}\left(\frac{\text{L}}{\text{L-b}}\right)$ $\leq$Allowable strength (S) | | | | | **(S7)** |
| --- | --- | --- | --- | --- | --- |
| Where | | | |  | |
| $\sigma$ | | **:** | | Actual stress. | |
| M_t_ | | **:** | | Torsional moment =$\frac{\text{H}}{\text{ω}}$. | |
| m | | **:** | | The module based on the largest tooth cross section $=D_{m}/Ng$= 3.55 | |
| D_m_ | | **:** | | Mean diameter of gear = r_m_ (gear). | |
| b | | **:** | | The face width of the gear = 30 mm. | |
| Y | | **:** | | Form factor based on the formative number of teeth and the type of tooth profile = 0.126. | |
| N | | **:** | | Actual number of teeth on gear = 41. | |
| S | **:** | | Allowable strength = $\text{S}_{0}\left(\frac{6}{\text{6+V}}\right)$ | | |
| S_0_ | **:** | | The endurance limit of the gear material for released loading = 500 MP_a_ ($\frac{1}{3}$ ultimate strength). | | |
| V | **:** | | The pitch line velocity m·s^-1^ = D_m_ = | | |
| L | **:** | | The cone diameter of gear = $\frac{\text{R}}{\text{cosγ}}$. | | |
| R | **:** | | Outer radius of the gear = 75 mm. | | |

S_actual_ < S_allawable_ , therefore, the design is safe

**S.2‎.4 Design of the main shaft**

The diameter of the transmission shaft was calculated according to ASME equation. The bending moment, axial load, and the torque acting on shaft are calculated previously. By applying all information in ASME code equation, it can get the diameter of the shaft. Figure S9 shows the bending moment diagram for the main shaft.

**R_X2_ =392.74 N**

**R_X1_ =678.36 N**

**W_t_= 2974.1 N**

**74620 N**·**mm**

**207196 N**·**mm**

**(a)**

**(b)**

**F_axial_ =2F_1-Roller_ + W_a_**

**F_1-Roller_ = 2051.34 N**

**F_1-Roller_ = 2051.34 N**

**W_r_ = 1071.1 N**

**W_a_ = 156.6 N**

**190 mm**

**110 mm**

**(c)**

**X**

**Y**

**B. M. D.**

**Figure S9.** Loads acting on the main shaft and bending moments applied on it. (a) Shaft forces, (b) Bending moment around Z-axis, (c) Bending moment around X-axis.

As a result of the applied loads acting on shaft, the following loads must between into consideration during the design of it.

Axial load, F_axial_ = 2 $\times$ 2051.34 + ‎156.6‎ = 4259 N

Applied torque, T= 1194268 N·mm (at 60 rpm)

Maximum bending moment, Mb (max) =

Mb (max) = **220223** N‎•‎mm

By applying the (**ASME** code equation) for shaft design with the following data:

| $\text{K=}\frac{\text{d}_{\text{i}}}{\text{d}_{0}}\text{=}\frac{0}{\text{d}_{0}}\text{=0}$ |
| --- |
| K_t_ = 1.5 to 3.0 load suddenly applied (rotating shaft) |
| K_b_ = 2.0 to 3.0 load suddenly applied (rotating shaft) |
| S_allowable_ = 40 MP_a_ |
| $\alpha$= 1 for tensile loading |

Thus, the diameter of the shaft should be equal or more than 62 mm.

**S.2‎.5 Transmission unit**

The power transmitted from the electric motor to the machine through a belt-pulley system as shown in Figure S10.

**Figure S10.** Power transmission system.

| $\frac{\text{n}_{1}}{\text{n}_{3}}\text{=}\frac{\text{D}_{2}}{\text{D}_{1}}\text{×}\frac{41}{6}$ | | |  | **(S8)** |
| --- | --- | --- | --- | --- |
| Where; | | | | |
| n_1_ | **:** | Rotational speed of the electric motor = ‎1440 ‎rpm | | |
| n_2_ | **:** | Rotational speed of transmission shaft. | | |
| n_3_ | **:** | Rotational speed of the main shaft = (100, 80, 60) rpm. | | |
| D_1_ | **:** | Diameter of the motor pulley. | | |
| D_2_ | **:** | Diameter of the machine pulley. | | |
| S_2_ | **:** | Number of teeth in pinion. | | |
| S_3_ | **:** | Number of teeth in gear. | | |

The Table S3 shows rotational speed of Motor and the rotational speed of the shaft with the different diameter of the pulleys.

**Table S3. The speeds and diameter for the pulleys.**

| **Motor Pulley** | |  | **Driven Pulley** | |  | **Driven Pulley** | |  | **Main shaft** | |
| --- | --- | --- | --- | --- | --- | --- | --- | --- | --- | --- |
| **D_1_ (mm)** | **n_1_ (rpm)** |  | **D_2_**  **(mm)** | **n_2_ (rpm)** |  | **S2** | **n_2_ (rpm)** |  | **S3** | **n_3_ (rpm)** |
| 120 | 1440 |  | 253 | 683 |  | 6 | 683 |  | 41 | 100 |
| 100 | 1440 |  | 263 | 547 |  | 6 | 547 |  | 41 | 80 |
| 80 | 1440 |  | 281 | 410 |  | 6 | 410 |  | 41 | 60 |

**Belt Force:**

The power transmitted by a belt drive is a function of the belt tensions and belt speed. As known, both sides of the belt are under tight forces with different values. The relation between these tight forces is as follows (Khurmi & Gupta, 2005), (Figure S11):

| $\text{(F}_{1}\text{-F}_{2}\text{)}\frac{\text{d}_{1}}{2}\text{=T}_{1}$, $\frac{\text{F}_{1}}{\text{F}_{2}}\text{=e}^{\text{μα}}$  F_B_=F_1_+F_2_ | | | **(S9)** |
| --- | --- | --- | --- |
| Where; | | | |
| F_1_ | **:** | Belt tension in tight side, N. | |
| F_2_ | **:** | Belt tension in loose side, N. | |
| $\mu$ | **:** | Coefficient of friction between belt and pulley (0.25). | |
| $\alpha$ | **:** | Angle of wrap of belt on pulley, rad. | |
| T_1_ | **:** | $\text{T}_{1}\text{=}\frac{\text{power}}{\text{ω}}$. | |
| F_B_ | : | Belt total force. | |

**Figure S11. Forces on belt pulley.**

Angles of Wrap ($\beta$) is the contact angle between pulley and the belt. It is calculated from the following relation:

| $\text{sinβ=}\frac{\text{R-r}}{\text{C}}$ | | | **(S10)** |
| --- | --- | --- | --- |
| $\alpha_{1}$ | : | (Small pulley) $\text{180-2β=180-2sin}^{\text{-1}}\frac{\text{R-r}}{\text{C}}$(Radius) | |
| $\alpha_{2}$ | : | (big pulley) $\text{180+2β=180+2sin}^{\text{-1}}\frac{\text{R-r}}{\text{C}}$ | |

The transmitted torque by the pulley (M_t_) was estimated to be **1194.268** N·m. From Eqs. (S9, S10), F_1_and F_2_ were estimated and given in Table S4.

**Table S4. Values of F_1_ and F_2_.**

| **D_1_ (mm)** | **D_2_ (mm)** | **F_1_ (N)** | **F_2_ (N)** | **F_1_+F_2_ (N)** |
| --- | --- | --- | --- | --- |
| 120 | 253 | 10158.2 | 717.4 | 10875.63 |
| 100 | 263 | 9807.3 | 725.4 | 10532.67 |
| 80 | 281 | 9224.2 | 724.0 | 9948.19 |

**Belt Length:**

V-belt is used to transmit motion between the motor and transmission shaft. The cross section and the length of V-belts have standardized by ANSI in U.S. customary units in SI unit. The length of V-belt is calculated from following equation, according to (Khurmi & Gupta, 2005).

| $\text{L=2C+1.57(D}_{2}\text{+D}_{1}\text{)+}\frac{\left(\text{D}_{2}\text{-D}_{1}\right)^{2}}{\text{4c}}$ | | | **(S11)** |
| --- | --- | --- | --- |
| Where; | |  | |
| L | **:** | Length of the belt | |

Belt length L = 150cm

**S.2‎.6 Electric motor**

The motor power is selected to the required power that calculated. The specification of the selected motor according is given in Table S5‎.

**Table S5. Specification of the used electric motor.**

| **Feature** | **Electric motor** |
| --- | --- |
| **Source of manufacture** | Germany |
| **Model** | AC. Electric motor |
| **Type** | SCHDRCH |
| **Speed, rpm** | 1440 |
| **Frequency, Hz** | 50 |
| **Power, kW** | 7.5 |
| **Vol. type, V** | 380 |
| **Current, A** | 15 |

**S.2‎.7 Frame and support**

The frame was manufactured locally from steel structures of angle-cross section (L2" ×2" ×1/8"). The dimensions of the frame are 10 cm length, 50 cm width, and 35 cm height. The frame includes two parts first part for supporting the motor and second part for supporting the machine.

**‎S.3 Finite Element Analysis (*FEA*)**

In this part, Finite Element analysis for the main parts of the pelleting machine, namely; main shaft and die will be discussed to analyze the stresses, strains and displacements that occur in these important parts of the pelleting machine.

**COSMOS / M** has been used as a FEA package. **COSMOS / M**, ranked one of the tremendous range of analysis capabilities, including:

1- Modeling, meshing and visualizing parts as well as assemblies.

2- Comprehensive analysis capabilities: stress, frequency, displacement, buckling, heat transfer nonlinear, dynamic response and fatigue capabilities.

3- Design optimization.

One of the basic capabilities features of **COSMOS / M** package is the static analysis. Static analysis is an extensive library of 1D, 2D and 3D elements that support isotropic, orthotropic, anisotropic, multi-layer composite and temperature dependent material properties. The geometry of the problem has to be constructed with the help of any computer aided design package.

**S.3.1 Material of construction**

Selection of materials for the main shaft and the die must be carefully considered where; it needs good knowledge of the mechanical properties of the material and its fabrication characteristics.

The selected material of these parts is heat treated to enhance its which properties. Where, it is oil quenched by a tempering process to obtain a crystal of high strength and little brittleness. The quenched process is obtained by heating these steel parts up to 950 ^o^C and then quenched in oil for low-rate of cooling. The quenching process as followed by a tempering process by heating the quenched parts up to 650 ^o^C and then cooled with a low rate of cooling (inside the furnace).

The mechanical properties of the used material (steel 50) are listed in **Table S6**. **(**Khurmi & Gupta, 2005**)**.

**Table S6. Mechanical properties of selected material.**

| **Mechanical property** | **Steel (St 50.11^*^)** |
| --- | --- |
| **Ultimate strength S_ut_ (MPa)** | 550 |
| **Yield strength S_y_ (MPa)** | 250 |
| **Young's modulus E_x_ (GPa)** | 200 |
| **Poisson ratio $\upsilon$_xy_** | 0.3 |
| **Shear modulus G_xy_ (GPa)** | 79 |
| **Density ρ (g /cm^3^)** | 7.7 |

*** Extract from DIN 1611&1612**

**‎**

**S.3.2 Numerical modeling of the main shaft of the pelleting machine**

The FEA analysis starts by defining some variables which are necessary for the analysis. These variables are: a) Type of the used element, b) Boundary conditions (Displacement attributes, loading attributes) and c) Stress and displacement results.

The input power to the shaft is delivered to shaft through section No. 3 and delivered out from the shaft to the rollers through sections No. 8 &9, as shown in Figure S12‎. The shaft is supported at sections No. 1 and 5 on tapered roller bearings and supported at section 7 on a deep groove ball bearing.


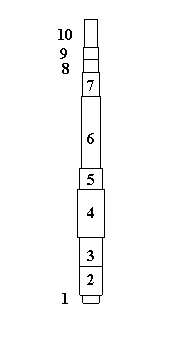


**Figure S12. Different sections of the main shaft.**

**S.3.2.1 Type of the used element group**

In this finite element analysis for the shaft, the reasonable element group used for this analysis is Plane 2-D with axisymmetric around Y-axis.

The PLANE 2D is a 4- to 8- node two dimensional element for plane stress or plane strain. All elements have to be defined in the X–Y axis plane. Axisymmetric structures have to be modeled in the positive X-axis half plane, in which X- axis represents the radial direction and Y-axis refers to the axis of symmetric for axisymmetric structures only two translational degrees of freedom per node are considered for structural analysis. In the axisymmetric, a one-radian sector is considered thus loads for a one-radian sector should be applied.

**S.3.2.2 Shaft modeling**

According to element group that chosen; the shaft was modeled in the **COSMOS/M** as a half part of the shaft. Y–axis represents the axis symmetry, as shown in Figure S13(a)‎. The half shaft was drawn through 29 points and 40 curves forming 10 surfaces, as shown in Figure S13(b).

**
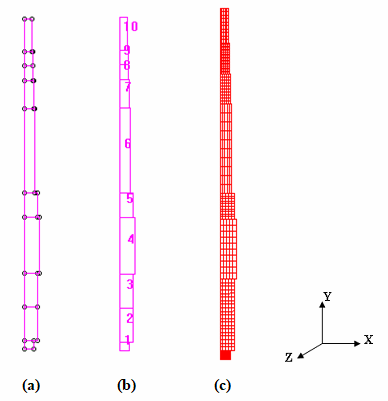

Figure S13.** Modeling shaft in the COSMOS/M: (a) Representation of points & curves. (b) Representation of surfaces. (c) Main shaft modeling and meshing.

**S.3.2.3 Element meshing of the problem**

Forming the element of the FEA for the shaft is carried out by meshing all surfaces of the model through the auto-mesh of the program. Each surface is meshed by 10 divisions in Y-direction 10 and 5 division in X-direction forming 500 elements and 660 nodes, as shown in Figure S13(c).

**S.3.2‎.4 Boundary conditions**

After shaft modeling, it is required to set the boundary conditions. The boundary conditions can be divided into two attributes, displacement attributes and loading attributes.

**S.3.2‎.4.1 Displacement attributes**

The displacement attributes show the shaft fixation and specify a displacement boundary condition value for a pattern of curves. The shaft is fixed on curves No. 4 (surface No. 1), No. 19 (surface No. 5) and No. 27 (surface No. 7), as shown in Figure S14‎. The degree of freedom of the point of curves No. 4 and No. 19 are:

U_X_ = U_Y_ =U_Z_ =0 (this means that are no translation degrees of freedom along global Cartesian X, Y, Z – Axes).

R_X_ = R_Z_ = 0 (That means, there are no rotational degrees of freedom about global Cartesian X and Z directions but has a rotational degrees of freedom about global Cartesian Y –Axis).

In displacement attribute on curve No. 27 (surface No. 7) has the following characteristics:

U_X_ =U_Z_ =0 (That means, that are no translational degrees of freedom along global Cartesian X and Z direction but it has translational degree of freedom along global Cartesian Y-axis).

R_X_ = R_Z_ = 0 (That means, that are no translational degrees of freedom along global Cartesian X and Z direction but it has translational degree of freedom along global Cartesian Y-axis).

**S.3.2‎.4.2 Loading attributes**

Loading attributes show and specify shaft loading types, direction and value for a pattern of surfaces. The prescribed value of the load is interpreted in the active coordinates system and should be non – zero value. In the present work, the shaft subjected to the following loads, Figure S15:

1- Tensile force: that acts on surface No. (8) due to the effect of the compaction rollers. So, the load set on the curve No. (31). The value of the force calculated as the one radian force for the section per unit length of these curves.

Total axial Force that resulting from the two rollers = 4102.68 N ‎

2- Torsional load: the input torque delivered to shaft through curve No. 11 (surface No. 3) and delivered out through curve No. 31 (surface No. 8 and 9). This torque is ‎258.5 N•m

The solution carried out as the linear static analysis.


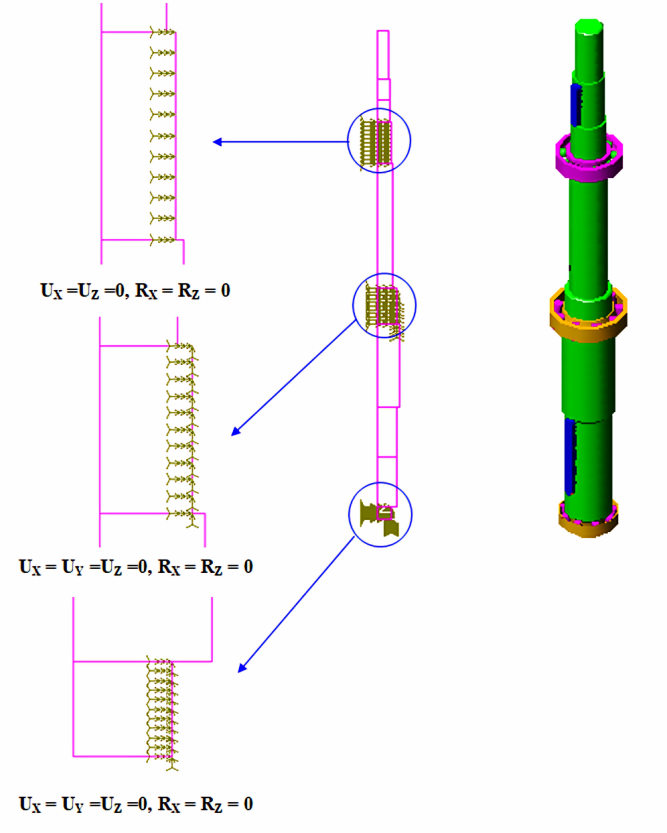


**Figure S14. Boundary conditions of the main shaft (displacement attributes).**

**
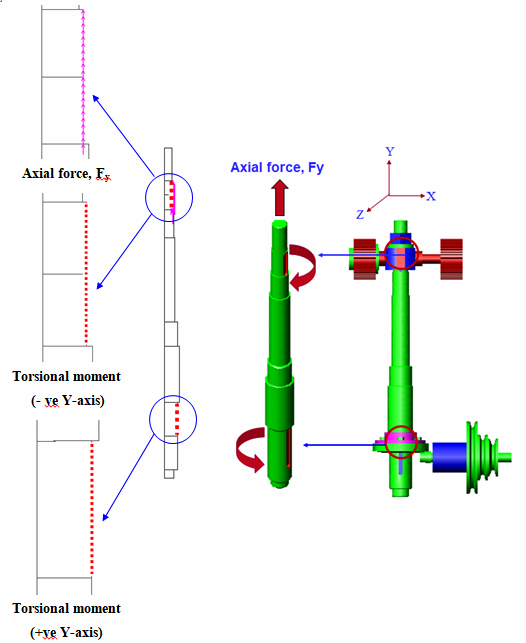
**

**Figure S15.** Boundary conditions of the main shaft (load attributes).

**S.3.2‎.5 Results**

**S.3.2‎.5.1 Stresses**

Throughout this numerical analysis, the von-Mises stress is obtained and used as a comparable stress. This stress is due to the applied tensile force and the torsional moments. From this numerical analysis, the maximum von-Mises stress is found to be in the range from **0.12** MPa to **32.54** MPa. Figure S20 shows the stress distributions along the shaft. From that Figure S16‎**,** the maximum stress occurs in elements No. (351) and (356) (surface No. 10). It can be observed that the maximum stress does not exceed 40 MPa, which reflects the design safety of the main shaft because the design calculation is performed based on 40 MPa as an allowable stress.


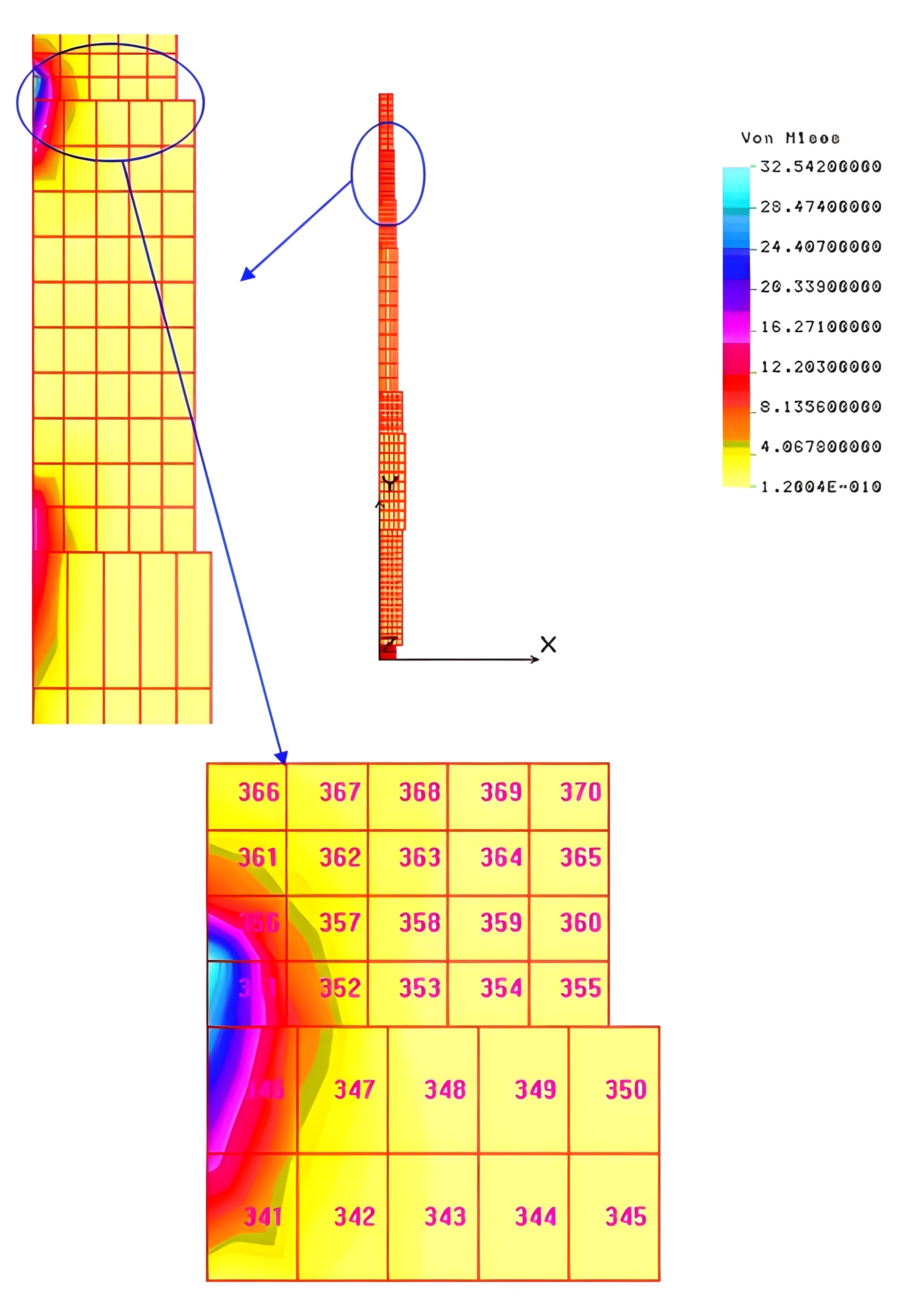


**Figure S16.** von-Mises stress (MPa) in the critical zone of the main shaft.

**S.3.2‎.5.2 Strain**

The maximum strains induced in the shaft due to the applied stresses in the shaft is found to be **9.5 × 10^-5^**. Figure S17 shows the strain distribution along the half shaft. It can be observed that the maximum strain is in element No. (351) (surface No. 9). This low induced strain in the shaft reflects the high rigidity of shaft which means that this shaft will be tough and stiff during its use in the pelleting machine.

**
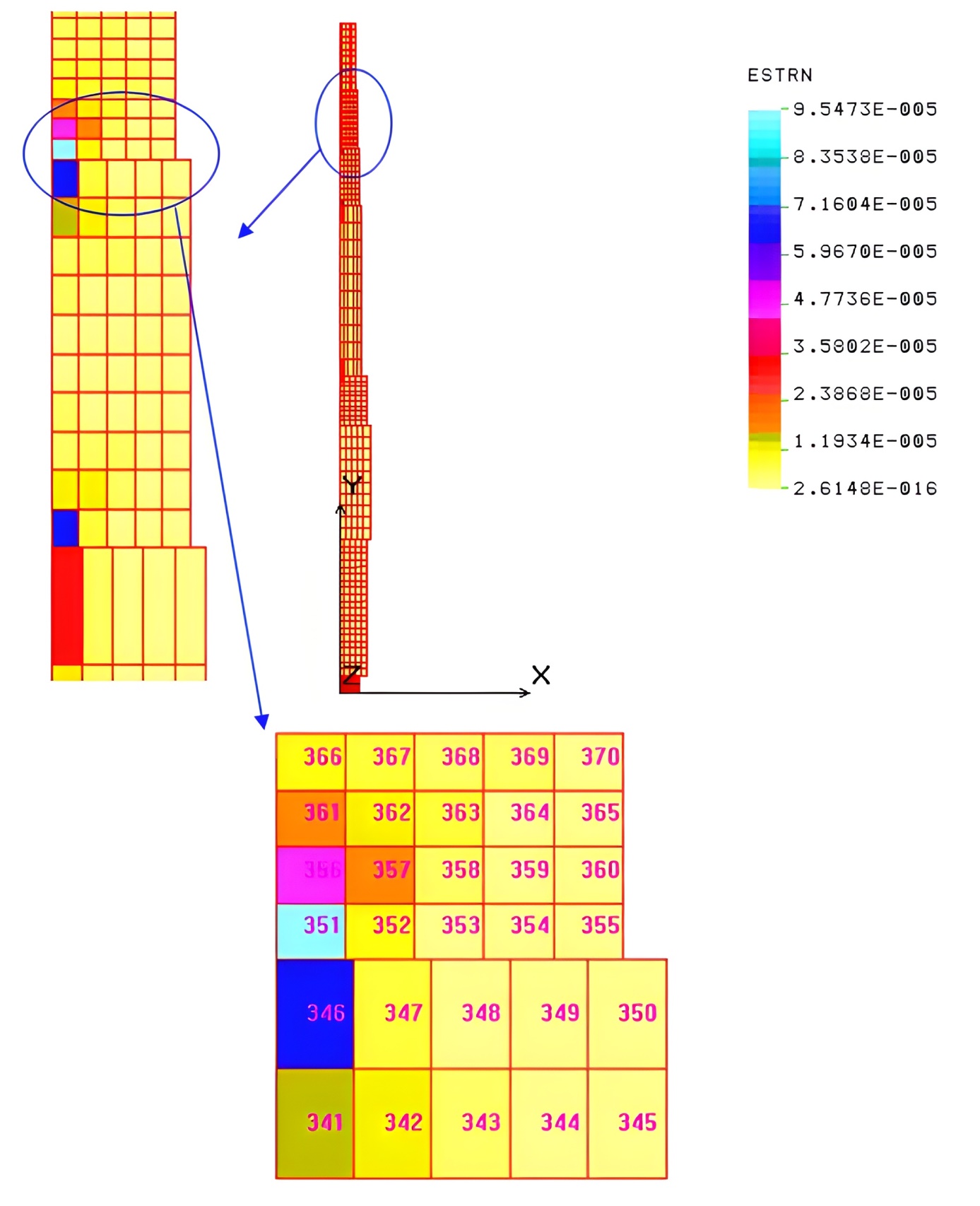
**

**Figure S17.** Strain distribution in the critical zone of the main shaft.‎

**S.3.2‎.5.3 Displacement and deformation**

Displacement of the different nodded of the shaft model is obtained as a results of the induced strains in the main shaft. From numerical analysis, the maximum displacement is found to be about **0.0053 mm**. Figure S22‎ shows the displacement distribution along the shaft. From that Figure S18, it can be observed that the maximum displacement is in element from (351) to (370) (surfaces No. (9) and (10)). This low displacement reflects the high rigidity of the shaft.


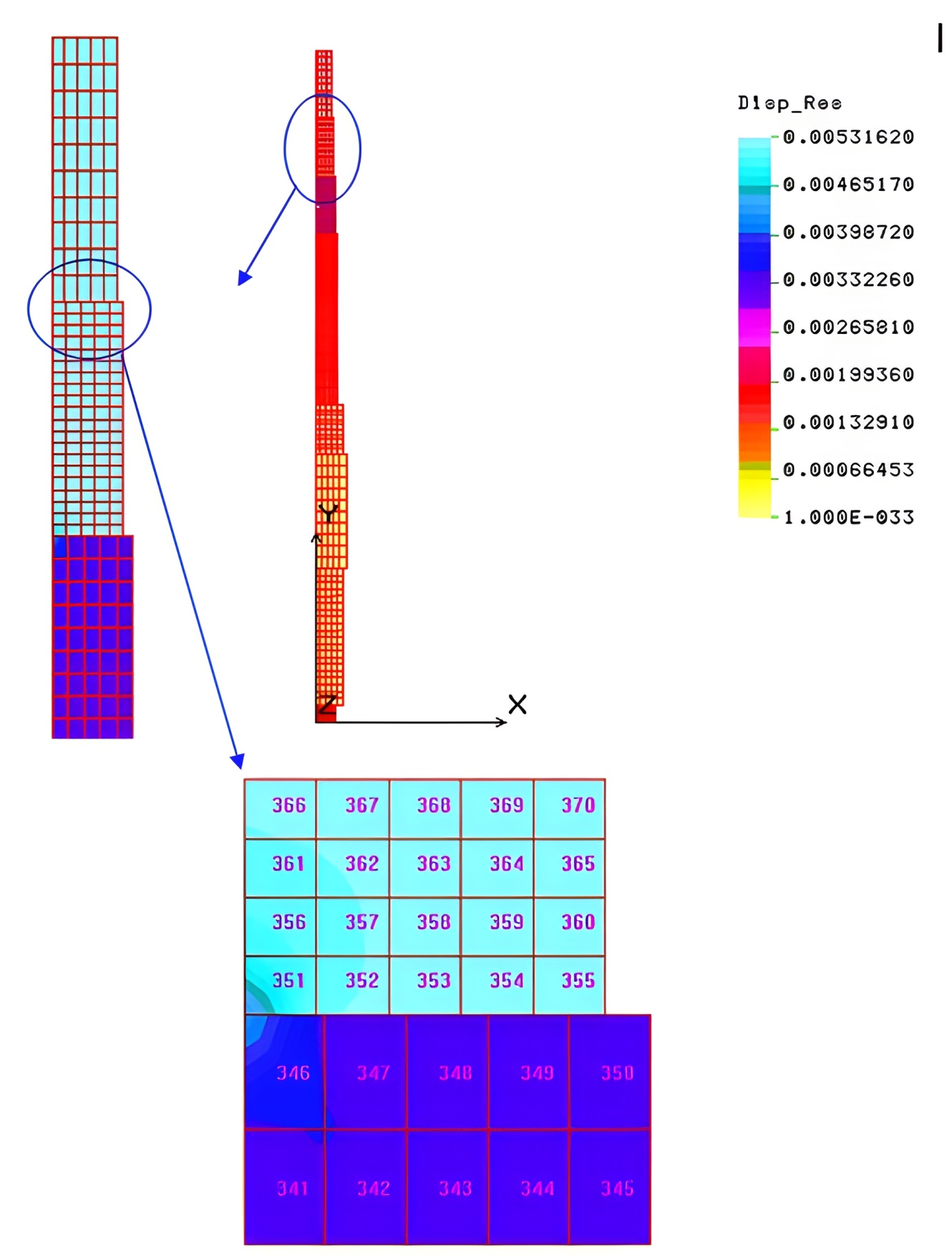


**Figure S18.** Displacement (mm) of the main shaft.‎

**S.3.3‎ Numerical analysis of the Die of the pelleting machine**

In this section, a finite element analysis for the die of the pelleting machine will be presented. The purposed of this analysis is to verify the theoretical design analysis results and to know the stress and strain distribution in the different die regions. The die has 136 extrusion holes (8 mm diameter) As shown in Figure S19. the holes of the die have counter 11 mm diameter and 25 mm depth As shown in Figure S19. The die before manipulation by **COSMOS/M,** it was taken to choice one extrusion hole to facilitate the studying the die.


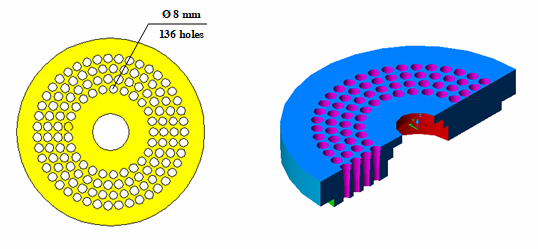


**Figure S19.** ‎ Geometry & sectional view ‎ of the die and distribution ‎of the holes

Each hole in the die has the following dimensions; inside diameter (ID) = 8 mm counter bored with Ø 11 mm and height (h) = 40 mm. The numerical analysis is performed for one of the die holes taken as a hollow cylinder having the same inside hole diameter of 8 mm counter bored with 11 mm in a depth of 25 mm as shown in Figure S20. The cylinder outside diameter is taken as the pith diameter between holes (=15mm).


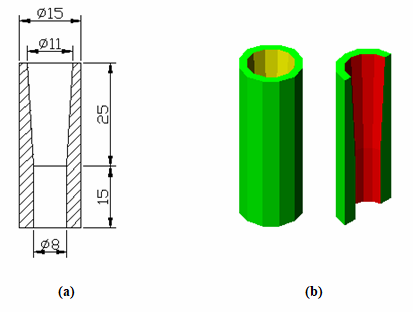


**Figure S20.** The one hole of the die (problem study): ‎(a) The dimension of the problem study, (b) 3 D of one hole and its suction‎

**S.3.3.1 Type of the used element group**

In this finite element problem, the element group used is a **SOLID** element. The **SOLID** is an 8- to 20-node three-dimensional element for the analysis of structural and thermal problems. Three translational degrees of freedom per node are considered for structural analysis. Only one degree of freedom per node, representing the temperature, is sued for the thermal module.

Both clockwise and counter-clockwise node numbering is allowed. Transitional elements can be considered by issuing zeros (0) at the location of missing nodes during the element connectivity definition. Prism and tetrahedron-shaped elements may be considered only with the 8-node element option. Prism-shaped elements may be formed by duplicating nodes (3 and 4) and (7 and 8). Pyramid elements are obtained if nodes 5, 6, 7 and 8 have the same global node number.

**S.3.3.2 Die modeling**

According to element group that chosen; The represented hole modeled in the **COSMOS/M**. Figure S21.‎ shows the modeling problem in 2 D and 3 D shape. After modeling the problem, the shape has 27 points, 52 curves and 36 surfaces forming 8 volumes.

**
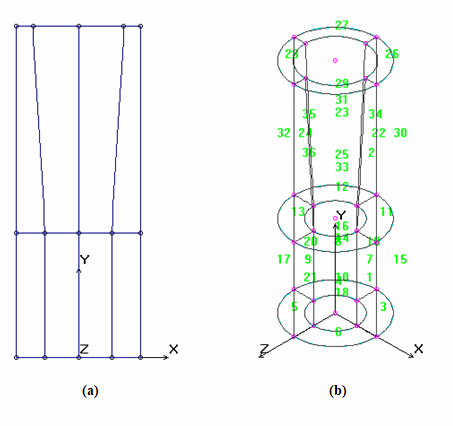
**

**Figure S21‎.** Modeling one hole of the extrusion die: ‎(a) The problem study in X-Y plane (2 D).‎ ‎(b) The problem study in X-Y-Z (3 D), and No. of the surfaces.‎

**S.3.3.3 Element meshing of the problem**

Forming the element of the FEA for the one hole of the die carried out by meshing all the volume of the model through the auto-mesh of the program. Each volume has 8 nodes where it is meshed by 5 divisions in Y-direction, 5 divisions in circumference of the circle and 4 divisions in diametrical direction forming 100. The result, the one hole of the die has 800 elements and 1410 nodes, as shown in Figure S22‎.

**
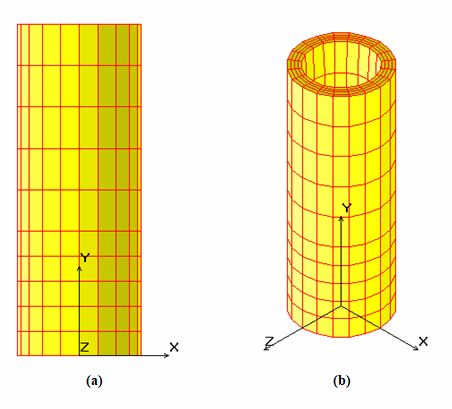
**

**Figure S22‎.‎** One hole of the die modeling after meshing: (a) The problem study in X-Y plan (2 D).‎ (b) The problem study in X-Y-Z (3 D).‎

**S.3.3.4 Boundary conditions**

After the die modeling, it is required to set the boundary conditions for die. The boundary conditions can be divided into two attributes, displacement attributes and loading attributes.

**S.3.3.4.1 Displacement attributes**

Figure S23‎ shows the one hole of the die fixation and specify a displacement boundary condition value for a pattern of surfaces. In the hole is fixed on surfaces No. (15) to No. (18) and on surfaces No. (30) to No. (33). The fixed on these surface has displacement attribute as the following characteristics:

- U_X_ = U_Y_ =U_Z_ =0 (that mean there is no translation degree of freedom along global Cartesian X, Y, Z – direction.

- R_X_ = R_Y_ = R_Z_ = 0 (That means, there is no rotational degree of freedom about global Cartesian X, Y, Z -direction.

**
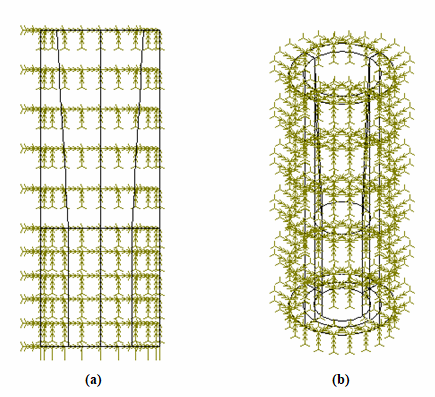
**

**Figure S23‎.** Boundary condition of the displacement: (a) The problem study in X-Y plan (2 D). (b) The problem study in X-Y-Z (3 D).‎

**S.3.3.4.2 Loading attributes**

Loading attributes show and specify shaft loading types, direction and value for a pattern of surfaces. The prescribed value of the load is interpreted in the active coordinates system and should be non – zero value. In the present work, the load is put as the pressure; the die is subjected to the following pressure: 1) Pressure subjected on the top of the hole, as shown in Figure S28. The pressure value is ‎145.18 MPa (From the calculation Hertzian equation). The pressure subjected on the surfaces No. (26) to No. (29). And 2) Pressure subjected on the internal hole, as shown in Figure S24. The pressure subjected on the surfaces No. (7) to No. (10) and on the surfaces No. (22) to No. (25).


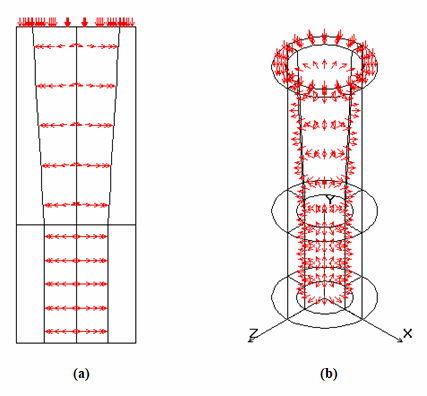


**Figure S24‎.‎** Boundary condition of load (pressure): ‎(a) The problem study in X-Y plan (2 D).‎ ‎(b) The problem study in X-Y-Z (3 D).‎

The solution of the problem is carried out as a linear static analysis.

**S.3.3.5 Results**

After programming running the result can be taken. It can be shown stress, strain, displacement and deformed shape of the shaft.

**S.3.3.5.1 Stresses**

Throughout this numerical analysis, the von-Mises stress is obtained as a comparable stress. This stress is due to the applied pressure from the input power to output power. Therefore, from numerical analysis the maximum von-Mises stress varied from **1.3 MPa** to **60.96** **MPa**. Figure S25 shows the stress distributions along one hole of the die. From that figure.**,** the maximum stress in the elements around the outside of one hole. They not exceed **60.96** **MPa**, which reflects the design safety of the die because after the harding of the die and tempering, the ultimate strength becomes **120 MPa**.


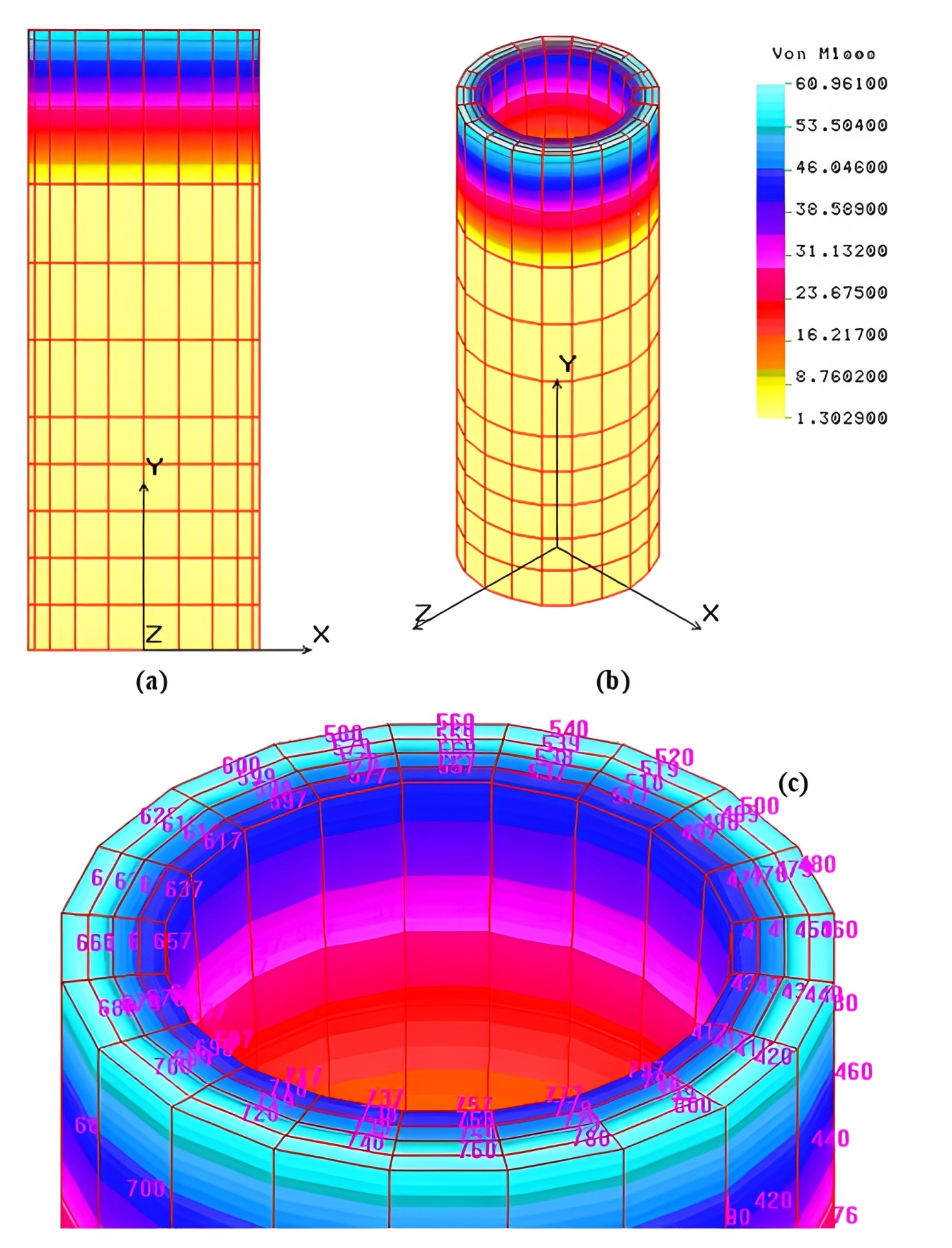


**Figure S25‎.** von-Mises stress (MPa) of one hole of the die: ‎(a) 2 D (b) 3 D (c) Maximum stress part‎

**S.3.3.5.2 Strain**

The maximum strains induced in the one hole due to the applied pressure on one hole of the die. Therefore, from numerical analysis, the maximum strain is **2.4 × 10^-4^**. Figure S26‎ shows the strain distribution along one hole of the die. This low induced strain in hole die reflects the high rigidity of shaft which means that this die will be tough and stiff during its use in the pelleting machine.


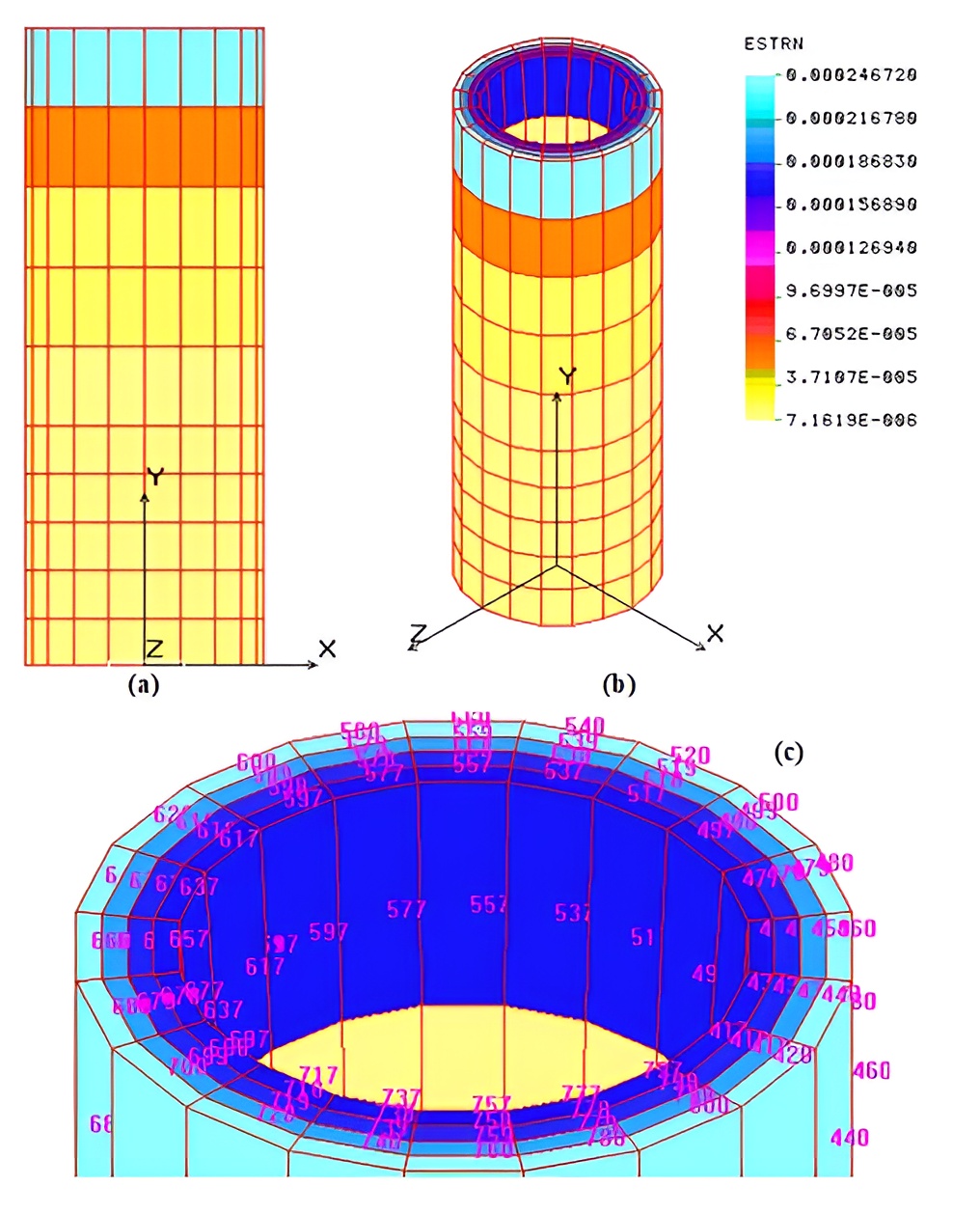


**Figure S26‎**.‎ Strain distribution of one hole of the die: ‎(a) 2 D (b): 3 D (c): Maximum strain part.‎

**S.3.3.5.3 Displacement**

The displacement of the different nodes of the hole is obtained as a results of the induced strain in the one hole of the die. This displacement is due to the applied pressure. From numerical analysis the maximum displacement is **0.001 mm**. This low displacement reflects the high rigidity of the die. Figure S27‎ shows displacement distribution along one hole of the die. From that Figure S31‎ it can be observed that the maximum displacement is around the outside of the hole.

**
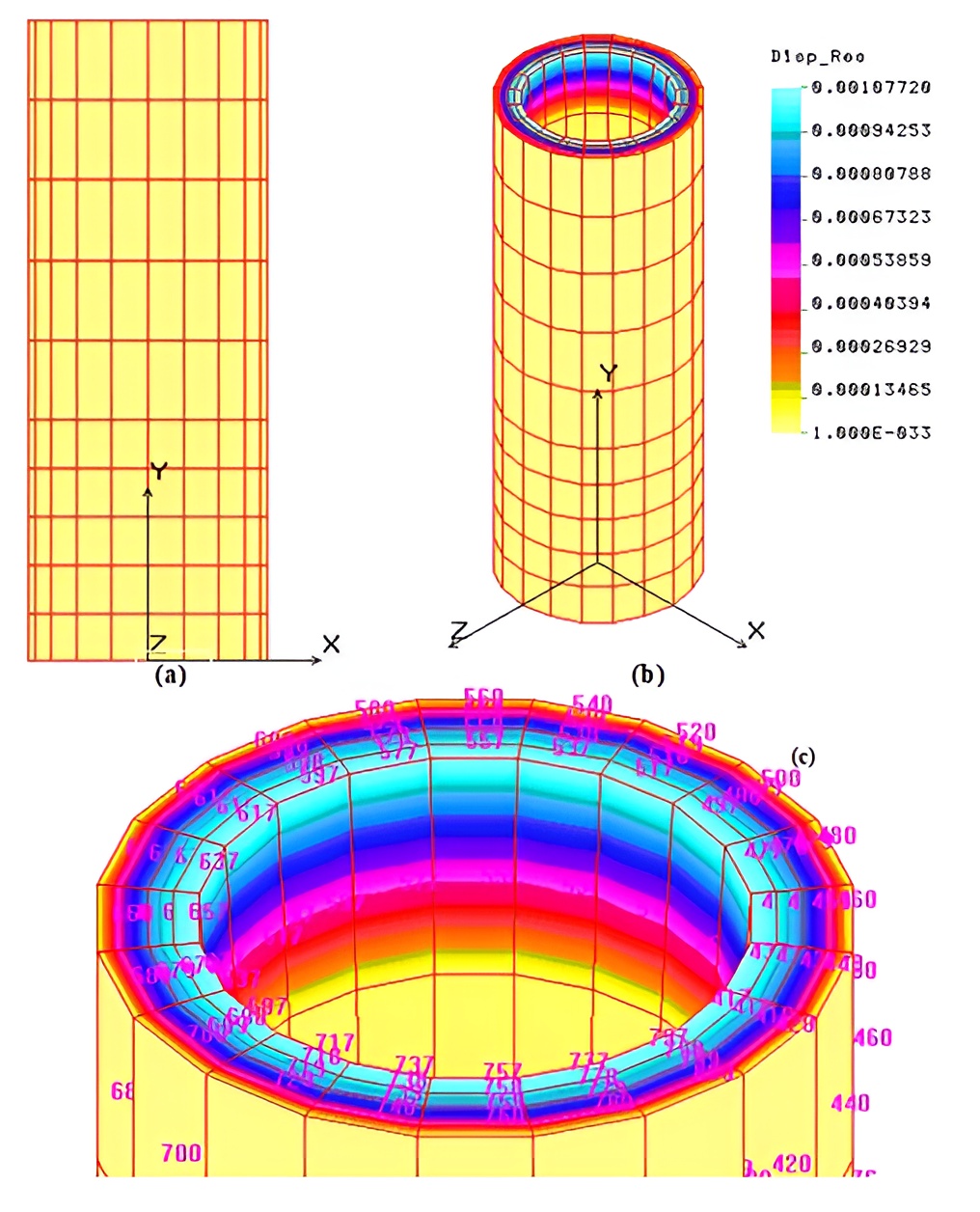
**

**Figure S27‎.‎** Displacement distribution (mm) of one hole of the die: (a) 2 D. (b) 3 D. (c) Maximum displacement part.

**S.3.3.5.4 Deformation of the die**

Figure S28‎ shows the one hole of the die after deformation. The scale factor of deformed shape about 2000 times that mean, the original deformed smaller than in that Fig. about 2000 times.


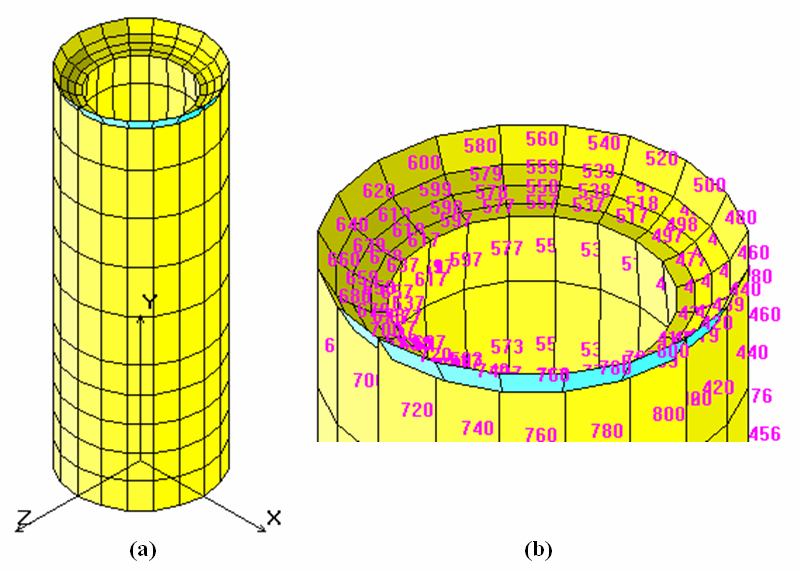


**Figure S28‎.** Deformed shape of one hole of the die: (a) The deformed shape. (b) Zooming the maximum deformed zone.‎

**S.4 Technical Drawing of the pelleting machine**

Figure S29 presents the technical representations of the designed pelleting machine. The first image displays a detailed 2D technical drawing, illustrating the internal components, assembly, and critical dimensions necessary for manufacturing. It includes sectional views that highlight key structural elements such as the main shaft, die, and transmission unit. The second image provides a real-world depiction of the fabricated machine, showing its physical construction and assembly. The structural frame, cylindrical housing, and material inlet are clearly visible, demonstrating the transition from design to implementation.

Figure S30 shows ‎the image showcases the fully assembled pelleting machine after fabrication. The machine features a sturdy metal frame providing structural support, with a cylindrical housing enclosing the internal components. The inlet for raw material is visible on the side, facilitating the feeding process. The power transmission system, including the pulley mechanism, is positioned on the side to drive the internal components efficiently. The machine's robust construction and bright red coating ensure durability and protection against external factors, making it suitable for industrial applications.


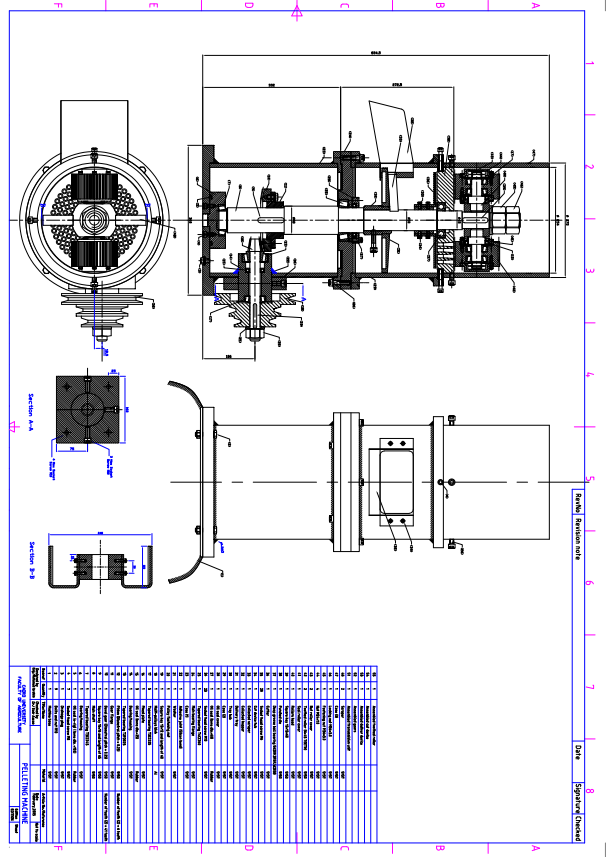


**Figure S29. Technical Drawing : 2D Representations of the Machine.**


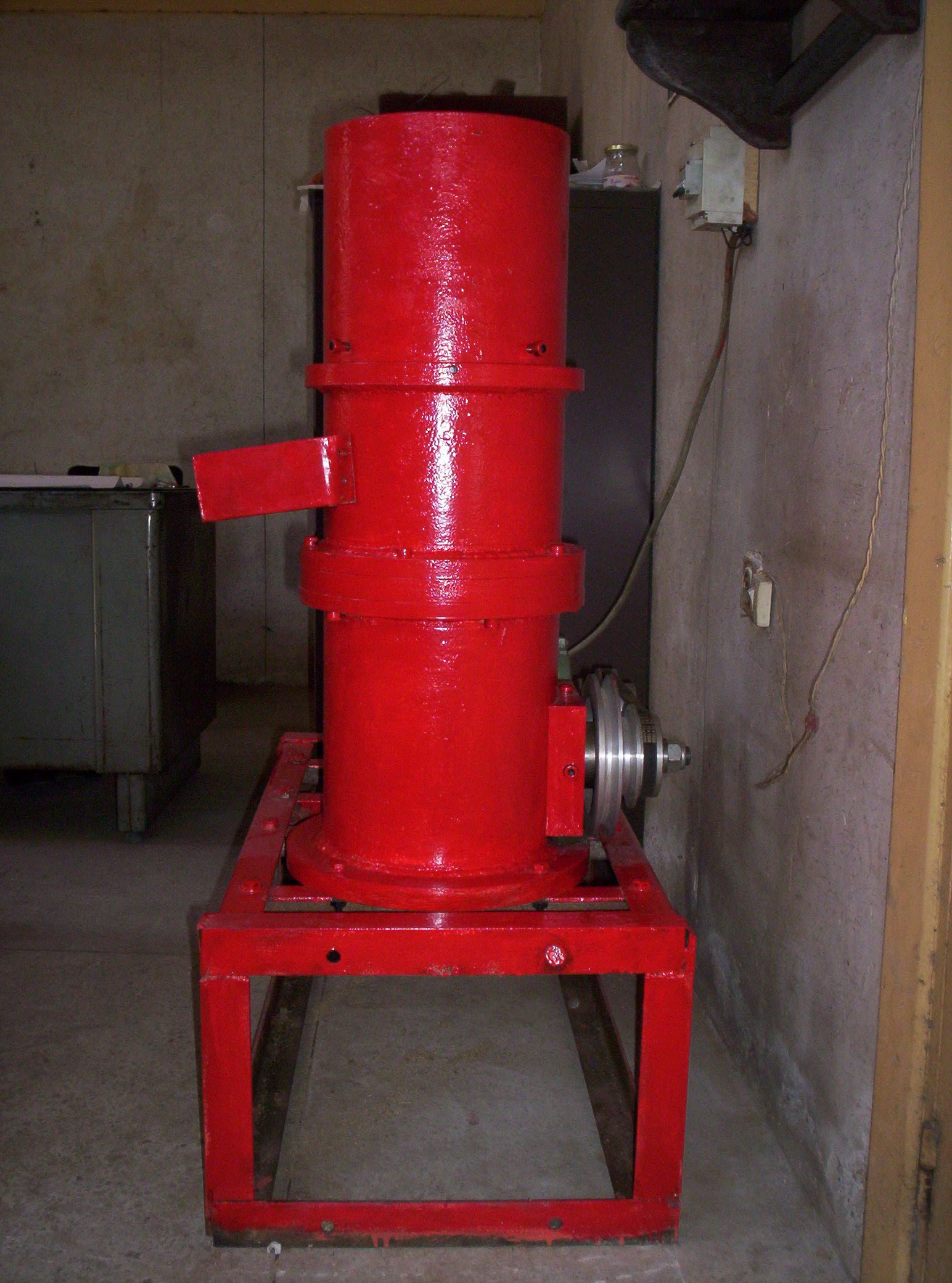


**Figure S30. Photograph of the Machine.**

References

Abdelbary, A., & Chang, L. (2023). *Principles of engineering tribology: Fundamentals and applications*. Elsevier.

Grote, K.-H., & Hefazi, H. (2021). *Springer handbook of mechanical engineering*. Springer Nature. <https://doi.org/https://doi.org/10.1007/978-3-030-47035-7>‎

Khurmi, R., & Gupta, J. (2005). *A textbook of machine design*. S. Chand publishing. <https://doi.org/https://doi.org/10.2307/3660180>

Lavergne, S., Larsson, S. H., Perez, D. D. S., Marchand, M., Campargue, M., & Dupont, C. (2021). Effect of process parameters and biomass composition on flat-die pellet production from underexploited forest and agricultural biomass. *Fuel*, *302*, 121076. <https://doi.org/https://doi.org/10.1016/j.fuel.2021.121076>‎

Semenov, A., Fomina, O., Muranov, A., Kutsbakh, A., & Semenov, B. (2019). The modern market of blank productions in mechanical engineering and the problem of standardization of new materials and technological processes. *Advanced materials & technologies*(1 (13)), 3-11. <https://doi.org/https://doi.org/10.17277/amt.2019.01.pp.003-011>‎

Standard, A. (2002). Cubes, Pellets, and Crumbles-Definitions and Methods for Determining Density, Durability, and Moisture Content. *American Society of Agricultural and Biological Engineers*.

Sun, W., Wang, Y., He, H., & Sun, Y. (2023). Compression prediction from single pellet press to industrial production presses. *Powder Technology*, *427*, 118719. <https://doi.org/https://doi.org/10.1016/j.powtec.2023.118719>‎

Uicker, J. J., Uicker Jr, J. J., Pennock, G. R., & Shigley, J. E. (2023). *Theory of machines and mechanisms*. Cambridge University Press. <https://doi.org/https://doi.org/10.1017/9781009303644>
